# A biomimetic gas-protein co-delivery nanoplatform targets mitochondrial stress and GSDME activation for glioblastoma immunotherapy

**DOI:** 10.1016/j.mtbio.2026.103750

**Published:** 2026-10-12

**Authors:** Guangyu Ma, Junjie Li, Kai Wang, Dehong Yu, Jiale Li, Ming Guan, Fenglei Gao, Zhao Liu, Jun Lan

**Affiliations:** a Department of Laboratory Medicine, Huashan Hospital, Shanghai Medical College, Fudan University, Shanghai, 200040, China; b Department of Thyroid and Breast Surgery, The Affiliated Hospital of Xuzhou Medical University, Xuzhou, Jiangsu, 221002, China; c School of Pharmacy, Xuzhou Medical University, Xuzhou, Jiangsu, 221004, China; d The Affiliated Pizhou Hospital of Xuzhou Medical University, Jiangsu, 221399, China; e Department of Orthopaedic Surgery, Lishui Central Hospital and Fifth Affiliated Hospital of Wenzhou Medical University, Zhejiang, 323000, China; f Department of Neurosurgery, The Affiliated Huai'an Hospital of Xuzhou Medical University, The Second People's Hospital of Huai'an, Huai'an, 223002, China

**Keywords:** Glioblastoma, Pyroptosis, Nanomedicine, Gas therapy, Immunotherapy

## Abstract

Glioblastoma (GBM) remains one of the most aggressive primary brain malignancies, with the therapeutic efficacy of conventional treatments and cancer immunotherapy being severely hindered by the blood-brain barrier (BBB) and the highly immunosuppressive tumor microenvironment (TME). Although pyroptosis, an inflammatory form of programmed cell death, can elicit potent antitumor immunity through the release of damage-associated molecular patterns (DAMPs), the precise induction of pyroptosis and the amplification of its immunostimulatory effects remain major challenges. Herein, we developed a biomimetic microglial cell membrane-coated nanosystem (M@GM) that integrates brain-targeted delivery, mitochondria-mediated damage, and innate immune activation for cascade-enhanced glioblastoma therapy. The nanosystem consists of a manganese carbonyl (MnCO) core loaded with Gasdermin E (GSDME) and cloaked with a microglial cell membrane, enabling efficient BBB penetration and active GBM targeting. Upon accumulation within the tumor microenvironment, M@GM responsively releases CO, Mn^2+^, and GSDME. CO and Mn^2+^ induced mitochondrial stress and ROS accumulation, which were accompanied by activation of the Caspase-3/GSDME axis and pyroptosis-associated cell death. The associated HMGB1 release and CRT exposure are consistent with an immunogenic cell death (ICD)-related response, while Mn^2+^ cooperatively activates the cGAS-STING innate immune pathway, promoting dendritic cell maturation, effector T-cell infiltration, and M1 polarization of tumor-associated macrophages, ultimately converting the immunologically “cold” GBM microenvironment into an immune-active “hot” phenotype. Both in vitro and in vivo studies demonstrated enhanced BBB transport and brain accumulation of M@GM, significantly inhibited GBM progression, prolonged the survival of tumor-bearing mice, and exhibited favorable biosafety. This work presents a biomimetic therapeutic strategy based on “biomimetic delivery-pyroptosis amplification-immune remodeling”, providing a promising design paradigm for brain-targeted nanomedicine-enabled cancer immunotherapy.

## Graphical abstract

A biomimetic microglial cell membrane-coated nanosystem that integrates brain-targeted delivery, mitochondria-mediated damage, and innate immune activation for cascade-enhanced glioblastoma therapy.

**Figure:**
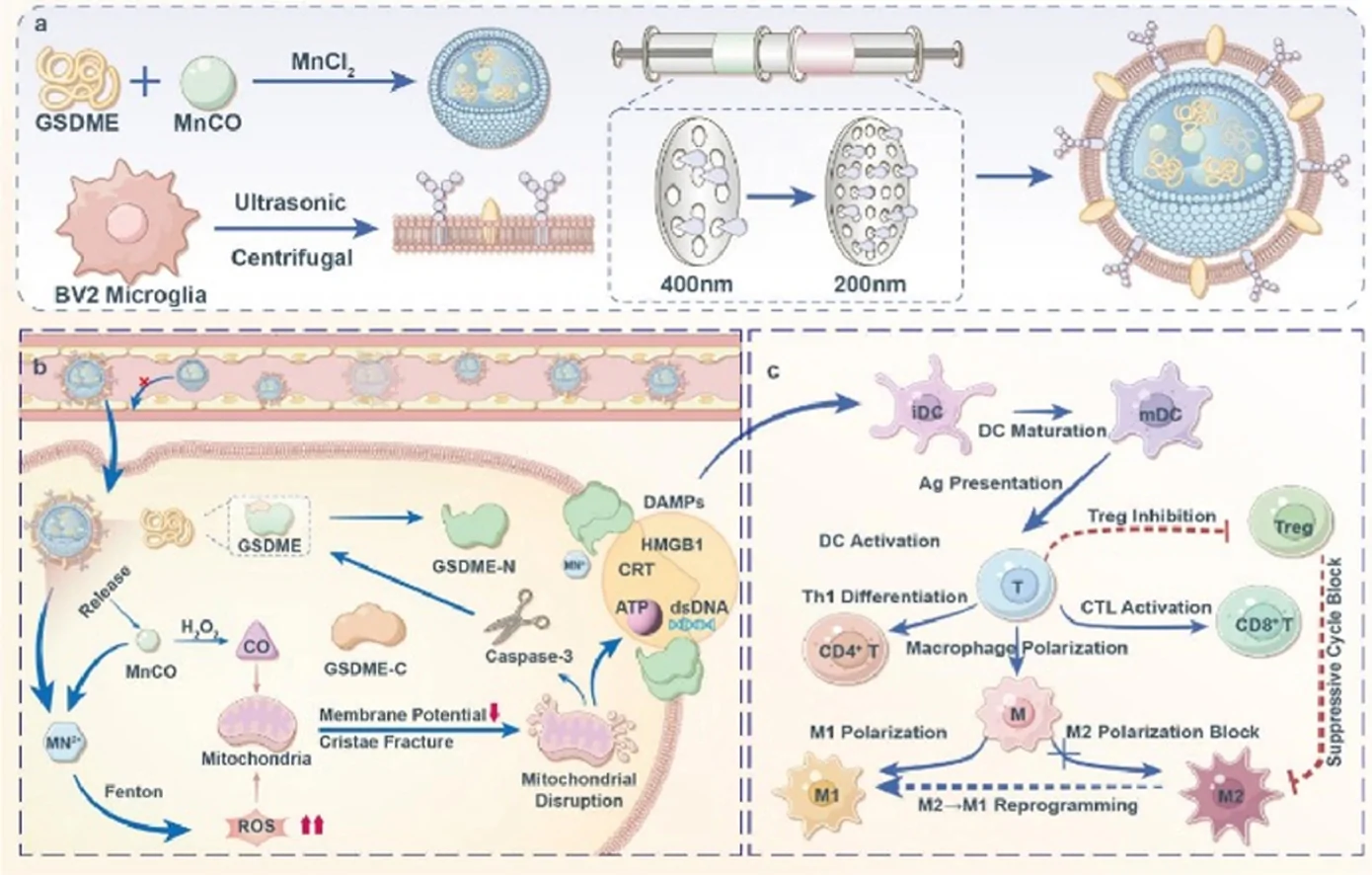

## 1 Introduction

Glioblastoma (GBM) is the most aggressive and lethal primary malignancy of the central nervous system [1], [2]. Although the current standard-of-care treatment consists of maximal surgical resection followed by radiotherapy and chemotherapy, successful treatment of GBM remains a formidable challenge because of the limited targeting efficiency of therapeutic agents and the severe side effects associated with conventional therapies [3], [4]. Moreover, the presence of the blood-brain barrier (BBB) severely restricts the efficient delivery of therapeutic agents into the brain parenchyma, while the highly immunosuppressive tumor microenvironment (TME) further compromises antitumor immune responses, rendering GBM a typical immunologically “cold” tumor [5], [6]. Therefore, the development of therapeutic strategies that simultaneously enable efficient brain-targeted drug delivery and potent immune activation is of great significance for improving the therapeutic outcomes of GBM [7] (see Scheme 1).

**Scheme 1:**
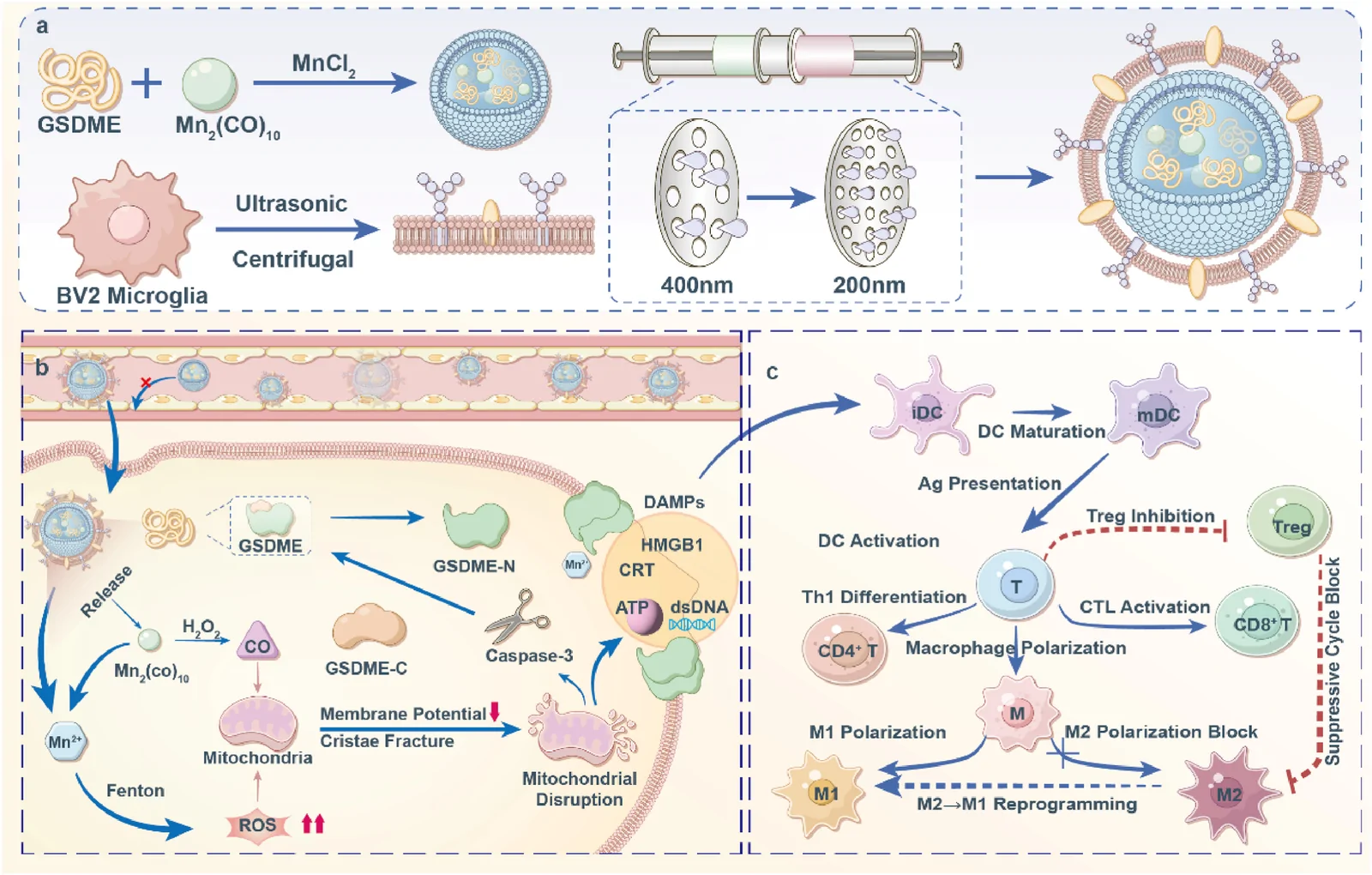
Schematic illustration of the construction of the biomimetic M@GM nanosystem and its cascade therapeutic mechanism for glioblastoma.

In recent years, pyroptosis, an inflammatory form of programmed cell death, has attracted increasing attention in cancer therapy [8], [9], [10]. Unlike apoptosis, pyroptosis not only directly induces tumor cell lysis but also promotes the release of abundant damage-associated molecular patterns (DAMPs), including high mobility group box 1 (HMGB1), adenosine triphosphate (ATP), calreticulin (CRT), and mitochondrial DNA (mtDNA). These endogenous danger signals subsequently trigger immunogenic cell death (ICD), promote dendritic cell maturation, and stimulate effector T-cell activation, thereby transforming local tumor cell death into a systemic antitumor immune response [11], [12], [13]. In particular, Gasdermin E (GSDME)-mediated pyroptosis has been demonstrated to markedly enhance immune surveillance and suppress tumor progression [14], [15], [16], [17]. Nevertheless, because pyroptosis is inefficiently activated in most tumor cells, achieving precise and efficient induction of GSDME-dependent pyroptosis remains a major challenge for cancer therapy.

Mitochondria, the central organelles responsible for cellular metabolism and redox homeostasis, are recognized as critical upstream regulators of pyroptosis [18]. Although carbon monoxide (CO) has traditionally been regarded as a toxic gas, accumulating evidence has demonstrated that low concentrations of CO can selectively target the mitochondrial respiratory chain, resulting in mitochondrial membrane depolarization, excessive reactive oxygen species (ROS) generation, and cellular stress responses, thereby activating the caspase cascade and subsequent cell pyroptosis [19], [20]. In addition, manganese ions (Mn^2+^) not only catalyze sustained ROS production through Fenton-like reactions, thereby aggravating mitochondrial dysfunction, but also function as potent innate immune agonists that significantly enhance the cyclic GMP-AMP synthase-stimulator of interferon genes (cGAS-STING) signaling pathway [21], [22]. Notably, the substantial release of mtDNA during pyroptosis further serves as an endogenous danger signal to activate the cGAS-STING pathway, suggesting the existence of a synergistic amplification network linking mitochondrial dysfunction, pyroptosis, and innate immune activation [18], [23]. However, the spatiotemporally coordinated delivery of CO, Mn^2+^, and pyroptosis-inducing effectors, together with the efficient amplification of their immunostimulatory activities, remains largely unexplored.

Inspired by the intrinsic glioma-homing capability of microglia, the resident immune cells of the central nervous system, biomimetic cell membrane-coated nanotechnology offers a promising strategy for overcoming the BBB and improving glioma-targeted drug delivery [24], [25], [26]. Microglial cell membranes not only preserve the native membrane protein composition and immune-evasive properties but also endow nanomedicines with enhanced brain penetration and tumor-targeting capabilities [27], [28]. Therefore, the construction of multifunctional biomimetic nanoplatforms camouflaged with microglial cell membranes holds great promise for simultaneously addressing the limited efficiency of brain drug delivery and the unsatisfactory efficacy of current immunotherapy.

Based on these considerations, we developed a biomimetic microglial cell membrane-coated nanosystem (M@GM), in which a manganese carbonyl (Mn_2_(CO)_10_) core was loaded with Gasdermin E (GSDME) and subsequently cloaked with a microglial cell membrane to facilitate BBB transport and enhance accumulation at GBM sites. Upon internalization by tumor cells, M@GM responsively releases CO, Mn^2+^, and GSDME within the tumor microenvironment. The released CO and Mn^2+^ are associated with mitochondrial dysfunction and enhanced ROS generation, thereby promoting activation of the Caspase-3/GSDME axis and enhancing pyroptosis-associated tumor-cell death. Meanwhile, mtDNA released during tumor-cell death may further contribute to cGAS-STING signaling, together with the immunomodulatory effects of Mn^2+^, promoting dendritic cell maturation, T-cell activation, and M1 polarization of tumor-associated macrophages, ultimately remodeling the glioblastoma immune microenvironment from an immunologically “cold” to a “hot” phenotype. Collectively, this study establishes a cascade therapeutic strategy integrating biomimetic delivery, mitochondria-mediated pyroptosis amplification, and immune remodeling, providing a promising theoretical basis and a novel design paradigm for brain-targeted nanomedicine-enabled cancer immunotherapy.

## 2 Materials and methods

### 2.1 Materials

Manganese carbonyl (CAS 10170-69-1, >99.9%), Recombinant GSDME protein (>95% purity), 2,7-Dichlorofluorescein diacetate (DCFH-DA), Calcein-AM, Propidium iodide (PI), Cell Counting Kit-8 (CCK-8), Phosphate-buffered saline (PBS), JC-1 staining kit, 4′,6-diamidino-2-phenylindole (DAPI), Annexin V-FITC/PI apoptosis detection kit, Dulbecco's Modified Eagle Medium (DMEM) and Roswell Park Memorial Institute Medium 1640 (RPMI-1640) were purchased from Key GEN Bio TECH Co., Ltd. (Nanjing, China). Bio Tracker Carbon Monoxide Probe 1 Live Cell Dye (SCT051, 1 mg) was purchased from Sigma Aldrich (Millipore Sigma, USA). Beyo Blue™ Plus Coomassie Brilliant Blue Ultra-Fast Staining Solution, ATP assay kit, membrane and cytoplasmic protein extraction kit were purchased from Beyotime Biotechnology Co., Ltd. (Nanjing, China). Polyclonal and monoclonal antibodies against Gasdermin E (GSDME), Gasdermin E N-terminal domain (GSDME-N), stimulator of interferon genes (STING), phosphorylated stimulator of interferon genes (p-STING) and interferon regulatory factor 3 (IRF3) were purchased from Thermo Fisher Scientific Inc. (Shanghai, China). Polyclonal antibodies against calreticulin (CRT) and high mobility group box 1 (HMGB1), as well as polyclonal and monoclonal antibodies against glyceraldehyde-3-phosphate dehydrogenase (GAPDH) and β-actin, were purchased from Proteintech Group (Wuhan, China).

### 2.2 Synthesis of GM nanoparticles

10 mg of manganese carbonyl powder was accurately weighed and dispersed in 15 mL of anhydrous methanol under argon protection, followed by ice-bath ultrasonication for 10 min 100W,3s on/2s off to form a uniform suspension [29]. GSDME recombinant protein solution (1 mg/mL) was slowly added to the suspension at an optimal protein-to-carrier mass ratio of 1:20, and the mixture was incubated at room temperature in the dark with gentle stirring for 1 h to allow sufficient protein adsorption onto the manganese carbonyl surface via electrostatic interactions. Subsequently, a slight excess of 1 mol/L MnCl_2_ solution was slowly added dropwise to the system, followed by vigorous stirring for 2 h to enhance metal ion coordination and preliminary assembly. Under continuous stirring, 1 mol/L NaOH solution was added dropwise to precisely adjust the pH to 11.0 ± 0.1, and the reaction was allowed to proceed at 25 °C with constant stirring for 4 h to complete nanoparticle crystallization. The product was centrifuged at 12,000 rpm for 10 min at 4 °C, and the precipitate was washed with ultrapure water 4-5 times to obtain GM nanoparticles.

### 2.3 Extraction and Purification of BV2 cell membranes

BV2 microglial cells at the logarithmic growth stage were collected and rinsed three times with ice-cold PBS. The cells were subsequently incubated on ice for 30 min in membrane extraction buffer containing a protease inhibitor mixture to facilitate membrane disruption. After mechanical homogenization by sonication, the lysate was sequentially centrifuged at 1000 × g for 10 min at 4 °C to eliminate nuclear fractions, followed by centrifugation at 10,000 × g for 20 min at 4 °C to remove intracellular organelles. The obtained supernatant was further subjected to ultracentrifugation at 10,000 × g for 1 h at 4 °C. The collected membrane pellet was resuspended in pre-chilled PBS and repeatedly washed twice through ultracentrifugation to obtain purified BV2 cell membranes. The membrane protein concentration was quantified using a bicinchoninic acid (BCA) protein assay kit and adjusted to 1-2 mg/mL. The purified membranes were aliquoted and stored at −80 °C until further application.

### 2.4 Preparation of M@GM nanoparticles

GM nanoparticles were resuspended in pre-cooled sterile ultrapure water at a concentration of 1 mg/ml. Freshly isolated BV2 microglial membrane suspension was added at a membrane protein-to-GM mass ratio of 1:3. The mixture was gently blended and incubated under slow rotation at 4 °C for 1 h, enabling the cell membrane to coat the nanoparticle surface via hydrophobic interaction. The mixture was centrifuged at 12000 rpm for 10 min at 4 °C, and the obtained precipitate was washed three times with pre-cooled sterile ultrapure water to acquire purified M@GM nanoparticles.

### 2.5 Cell lines and animals

Mouse glioblastoma cell line (GL261), mouse microglial cell line (BV2), mouse brain microvascular endothelial cell line (bEnd.3) were obtained from FuHeng Cell Center (Shanghai, China). All animal procedures were performed in compliance with protocols approved by the Institutional Animal Care and Use Committee of Xuzhou Medical University (Ethics Approval No.: 202512T014). Female C57BL/6J mice (4-6 weeks old) were purchased from the Laboratory Animal Center of Xuzhou Medical University (Xuzhou, China).

### 2.6 Cellular uptake assay of M@GM

M@GM nanoparticles were labeled with fluorescein isothiocyanate (FITC). Briefly, 1 mg of FITC dye was added to 5 mL of M@GM solution (5 μg/mL) and mixed thoroughly to obtain FITC-labeled nanoparticles. GL261 cells were seeded in confocal dishes at a density of 6 × 10^4^ cells/well and cultured for 24 h at 37 °C in a 5% CO_2_ incubator. The cells were treated with FITC-labeled M@GM and incubated for 0 h, 1 h, 2 h, 4 h, and 6 h, respectively. After incubation, the cells were washed three times with pre-cooled PBS at 4 °C, stained with DAPI for nuclear labeling, and washed again. Images were acquired by confocal laser scanning microscopy (CLSM), and quantitative fluorescence analysis was performed by flow cytometry (Becton Dickinson, USA).

### 2.7 In Vitro cytotoxicity

BV2, GL261, and U87 cells were seeded in 96-well plates at 8 × 10^3^ cells/well and cultured for 12 h. The cells were treated with M@GM at gradient concentrations of 0, 5, 10, 15, 20, 25, 30, and 35 μg/mL for 24 h. After washing three times with PBS, 100 μL of medium containing 10% CCK-8 was added to each well, and the plates were incubated at 37 °C for 12 h. The absorbance at 450 nm was measured by a microplate reader to calculate the cell viability of each group.

### 2.8 Live/dead staining

GL261 cells were seeded in 6-well plates at a density of 4 × 10^5^ cells/well and cultured overnight. The cells were divided into PBS, M, GM, and M@GM groups for corresponding drug treatment. After incubation, the cells were stained with PI/Calcein AM mixed staining solution in the dark for 30 min, and the cell viability was observed and recorded under an inverted fluorescence microscope.

### 2.9 Cell apoptosis detection

GL261 cells seeded in 6-well plates were treated with corresponding formulations. The cell suspension was collected, washed three times with pre-cooled PBS, and resuspended in Binding buffer to prepare a single-cell suspension. Annexin V-FITC and PI double staining reagents were added to the cell suspension, followed by incubation at room temperature in the dark for 15 min. The suspension was diluted with Binding buffer and detected by flow cytometry within 1 h to analyze the apoptosis rate.

### 2.10 Statistical analysis

All quantitative data are presented as mean ± standard deviation (SD). Statistical analyses were conducted using one-way analysis of variance (ANOVA) and Student's t-test to determine the statistical differences among experimental groups. A P value of < 0.05 was considered statistically significant. The levels of statistical significance were indicated as follows: *P < 0.05, **P < 0.01, ***P < 0.001, and ****P < 0.0001.

## 3 Results

### 3.1 Preparation and characterization of M@GM nanoparticles

As illustrated in Fig. 1a, the GM nanocore was first constructed using manganese carbonyl (Mn_2_(CO)_10_) and GSDME, followed by coating with BV2 microglial cell membranes to obtain the biomimetic nanosystem M@GM. TEM images showed that the GM nanoparticles exhibited a uniform spherical morphology with good monodispersity (Fig. 1b and c). After membrane coating, M@GM maintained a regular spherical morphology, and a distinct core-shell structure was clearly observed (Fig. 1d). Scanning electron microscopy (SEM) images further revealed that M@GM possessed a smooth surface morphology (Fig. S1), supporting successful membrane coating. Elemental mapping analysis demonstrated that the Mn signal was highly colocalized with the N, S, and P signals derived from the microglial cell membrane (Fig. 1e), supporting encapsulation of the manganese-based nanocore by the cell membrane. In addition, Coomassie Brilliant Blue staining showed that the protein band profile of M@GM was highly consistent with that of the native microglial cell membrane (Fig. 1f), indicating that the membrane-associated protein profile was retained after coating.

**Fig. 1:**
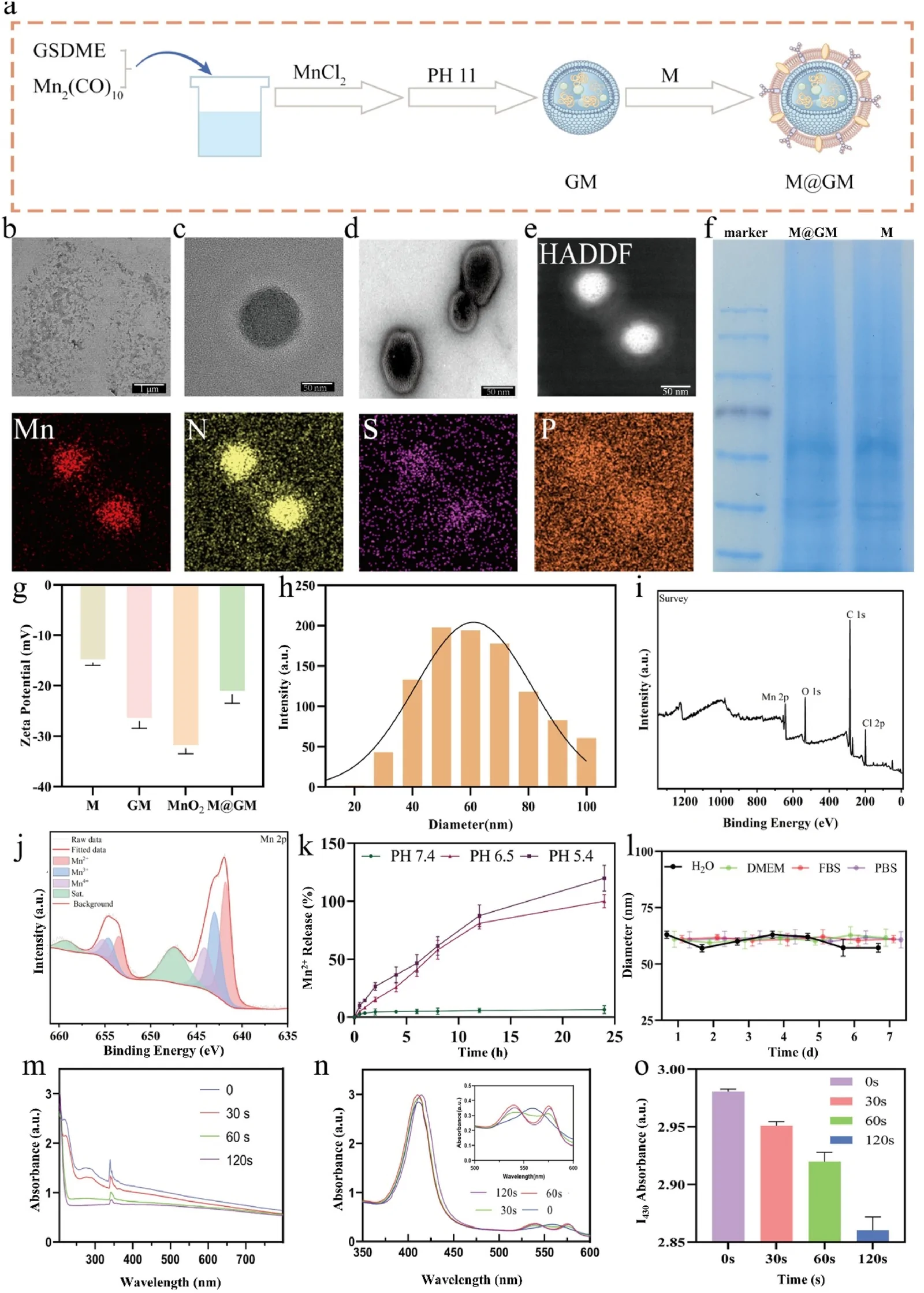
Fabrication and physicochemical characterization of the biomimetic M@GM nanosystem. (a) Schematic illustration of the preparation of microglial cell membrane-coated M@GM nanoparticles. (b, c) TEM images of GM nanoparticles at different magnifications. (d) TEM image of M@GM nanoparticles. Scale bars:1 μm. (e) HAADF-STEM image and corresponding EDS elemental mapping of Mn, N, S, and P in M@GM nanoparticles. Scale bars:50 nm. (f) Coomassie Brilliant Blue staining analysis of protein profiles from BV2 cell membranes and M@GM nanoparticles. (g) Zeta potential of GM, BV2 cell membrane, MnO_2_, and M@GM nanoparticles. (h) Hydrodynamic size distribution of M@GM. (i) XPS spectrum of M@GM. (j) XPS spectrum of Mn 2p. (k) pH-responsive Mn^2+^ release profiles of M@GM under different pH conditions. (l) Colloidal stability of M@GM after storage for 7 days. (m) UV-vis absorption spectra of M@GM after incubation with H_2_O_2_ for different durations. (n) Time-dependent UV-vis absorption spectra of Hb-CO and Mb-CO during incubation with M@GM. (o) Quantitative analysis of CO release from M@GM at different incubation times determined by the myoglobin assay.

Dynamic light scattering (DLS) analysis revealed that M@GM exhibited a narrow size distribution with a hydrodynamic diameter predominantly ranging from 40 to 80 nm and an average particle size of approximately 60 nm (Fig. 1h). Zeta potential measurements showed that the surface potential of M@GM was comparable to that of the native microglial cell membrane (Fig. 1g), consistent with substantial membrane coverage of the nanocore. X-ray photoelectron spectroscopy (XPS) analysis further confirmed the presence of characteristic Mn 2p, O 1s, C 1s, and Cl 2p peaks in the spectrum (Fig. 1i and j). The loading contents of GSDME and MnCO in M@GM were further quantified using a UV-vis spectrophotometric mass-balance method. Standard calibration curves were established for GSDME (Fig. S2a) and MnCO (Fig. S2b), and the unreacted components in the post-assembly supernatant were quantified according to their characteristic absorption signals (Fig. S2c). The amounts incorporated into M@GM were calculated by subtracting the unreacted amounts from the corresponding initial amounts. Accordingly, the loading contents of GSDME and MnCO were determined to be 7.2% and 11.0%, respectively. Collectively, these results demonstrate the successful fabrication of the biomimetic M@GM nanosystem with a well-defined core-shell architecture and preserved membrane protein composition.

Subsequently, the pH-responsive release behavior of M@GM was evaluated by measuring Mn^2+^ release in PBS at pH 6.5 and pH 5.5. M@GM rapidly degraded under acidic conditions while remaining stable at physiological pH. Under physiological conditions (pH 7.4), the relative Mn^2+^ release (normalized to the Mn^2+^ concentration after 24 h incubation at pH 6.5) remained negligible throughout the 24 h incubation period. In contrast, under acidic conditions simulating the tumor microenvironment (pH.

6.5) and lysosomal environment (pH 5.4), the relative Mn^2+^ release increased progressively with incubation time, and the release rate further accelerated as the pH decreased, demonstrating a time-dependent and acidity-responsive release behavior (Fig. 1k). In addition, M@GM remained well dispersed in various aqueous media and showed no obvious aggregation after 7 days of storage (Fig. 1l). The well-defined core-shell structure and preserved microglial membrane proteins provide an important structural basis for the subsequent BBB penetration and tumor-targeting properties of M@GM. Meanwhile, the co-loading of MnCO and GSDME enables coordinated delivery of the upstream therapeutic trigger and downstream pyroptotic effector within a single nanoplatform.

### 3.2 In Vitro degradation and CO release behavior of M@GM

Considering the highly oxidative nature of the tumor microenvironment (TME), the degradation behavior of M@GM in the presence of hydrogen peroxide (H_2_O_2_) was investigated using UV-vis absorption spectroscopy. As shown in Fig. 1m, the characteristic absorption intensity of M@GM gradually decreased with increasing H_2_O_2_ incubation time, indicating the progressive degradation of the nanoparticles under oxidative conditions.

The CO-releasing capability of M@GM was subsequently evaluated using the classical myoglobin assay [30]. Time-dependent UV-vis absorption spectra showed that, with prolonged incubation with M@GM, the characteristic absorption peaks of deoxy myoglobin (Hb-CO) gradually decreased at 529 and 557 nm, whereas the characteristic peaks of carbonmonoxy myoglobin (Mb-CO) at 540 and 570 nm progressively appeared and increased in intensity. These spectral changes confirmed the responsive release of CO from M@GM under simulated tumor microenvironment conditions. Quantitative analysis further demonstrated that the amount of released CO continuously increased with incubation time, and detectable CO release was observed as early as 120 s (Fig. 1n and o), indicating the rapid and time-dependent CO-releasing behavior of M@GM under TME-mimicking conditions.

To further mimic the complex tumor microenvironment, the release behaviors of Mn^2+^ and CO were investigated under combined acidic and oxidative conditions. CO release was enhanced under acidic conditions and further increased in the presence of H_2_O_2_, with the highest release observed under the combined acidic/H_2_O_2_ condition (Fig. S3a). Similarly, Mn^2+^ release was markedly promoted by H_2_O_2_ and further accelerated under acidic conditions, indicating the coordinated responsiveness of M@GM to both acidic and oxidative stimuli (Fig. S3b). The enhanced release of CO and Mn^2+^ under acidic and oxidative conditions enables M@GM to respond preferentially to the tumor microenvironment. This feature may help minimize premature activation during circulation while facilitating therapeutic payload release after tumor accumulation.

### 3.3 In Vitro BBB penetration capability of M@GM

To evaluate the blood-brain barrier (BBB) penetration capability of the microglial cell membrane-coated M@GM nanoparticles and their inhibitory effects on glioblastoma cell migration, an in vitro BBB model was established using a Transwell co-culture system. As illustrated in Fig. 2a, bEnd.3 brain endothelial cells were seeded onto the upper Transwell inserts to form a confluent monolayer, while GL261 glioblastoma cells were cultured in the lower chamber. The integrity of the BBB model was verified by measuring the trans endothelial electrical resistance (TEER). As shown in Fig. 2b, the resistance of the bEnd.3 monolayer increased markedly, indicating the formation of a tight endothelial barrier. The cellular uptake of nanoparticles by GL261 cells in the lower chamber was visualized after 0.5 h and 2 h of incubation. Fluorescence imaging and quantitative analysis demonstrated that the intracellular fluorescence intensity in the M@GM-treated group was significantly higher than that in the uncoated GM group (Fig. 2c, d and Fig. S4), indicating enhanced transport of M@GM across the in vitro BBB model. To further investigate the mechanism underlying the enhanced BBB transport of M@GM [31], [32], a VCAM-1 blocking experiment was performed using an anti-VCAM-1 antibody (Fig. S5). M@GM exhibited strong fluorescence signals after crossing the endothelial barrier at both 0.5 and 2 h, whereas VCAM-1 blockade markedly attenuated the translocation-associated fluorescence signals. These findings indicate that VCAM-1-mediated interactions contribute to the efficient BBB transport of M@GM.

**Fig. 2:**
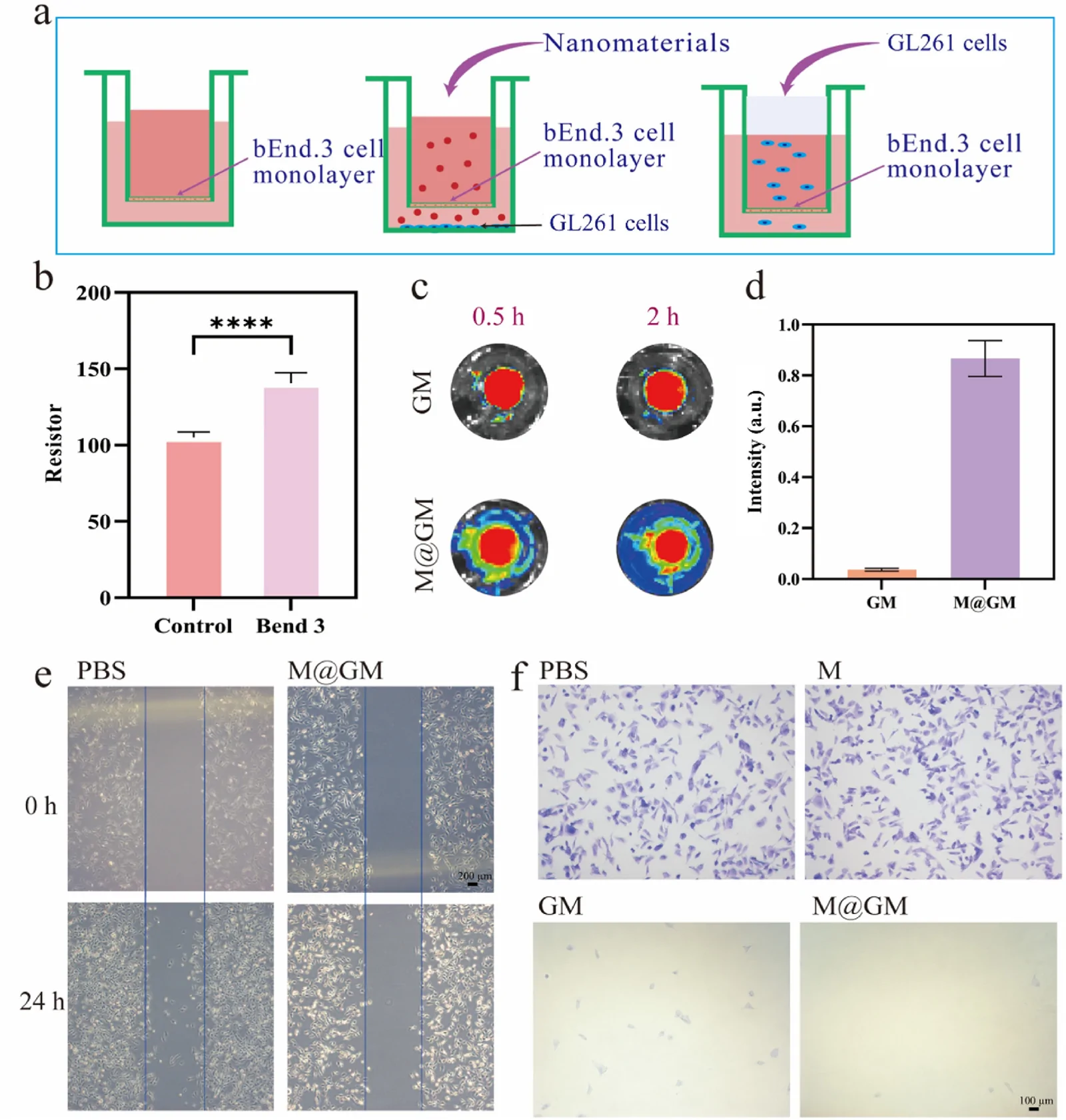
In vitro evaluation of the BBB penetration capability and anti-migratory activity of the biomimetic M@GM nanosystem. (a) Schematic illustration of the Transwell-based in vitro BBB model established using a bEnd.3 cell monolayer and GL261 glioblastoma cells. (b) TEER values of the bEnd.3 monolayer for evaluating BBB integrity. (c) Fluorescence images showing the transport and cellular uptake of GM and M@GM by glioblastoma cells after crossing the in vitro BBB model at 0.5 and 2 h. (d) Quantitative analysis of intracellular fluorescence intensity after nanoparticle transport across the BBB model. (e) Representative images of the wound healing assay after treatment with PBS and M@GM for 0 and 24 h. Scale bars:200 μm. (f) Representative images of the Transwell invasion assay for PBS, M, GM, and M@GM-treated glioblastoma cells. Scale bars:100 μm.

The effects of M@GM on the migratory and invasive behaviors of GL261 cells were further evaluated using wound healing and Transwell invasion assays. In the wound healing assay, the PBS-treated group exhibited substantial wound closure after 24 h, whereas a much wider wound gap remained in the M@GM-treated group, indicating reduced cell migration (Fig. 2e). Consistently, the Transwell invasion assay showed that M@GM treatment markedly decreased the invasive capacity of GL261 cells compared with the PBS, M, and uncoated GM groups (Fig. 2f).

### 3.4 In Vitro biosafety evaluation of M@GM

Efficient cellular internalization is essential for the therapeutic performance of M@GM. To investigate the cellular uptake behavior, GL261 cells were incubated with FITC-labeled M@GM for different time periods (0, 1, 2, 4, and 6 h), and nanoparticle internalization was analyzed by confocal laser scanning microscopy (CLSM) and flow cytometry. As shown in Fig. 3a, the intracellular FITC fluorescence gradually increased with prolonged incubation time, indicating the time-dependent uptake of M@GM by GL261 cells. Flow cytometric analysis further confirmed the progressive increase in intracellular nanoparticle accumulation over time (Fig. 3b and Fig. S6).

**Fig. 3:**
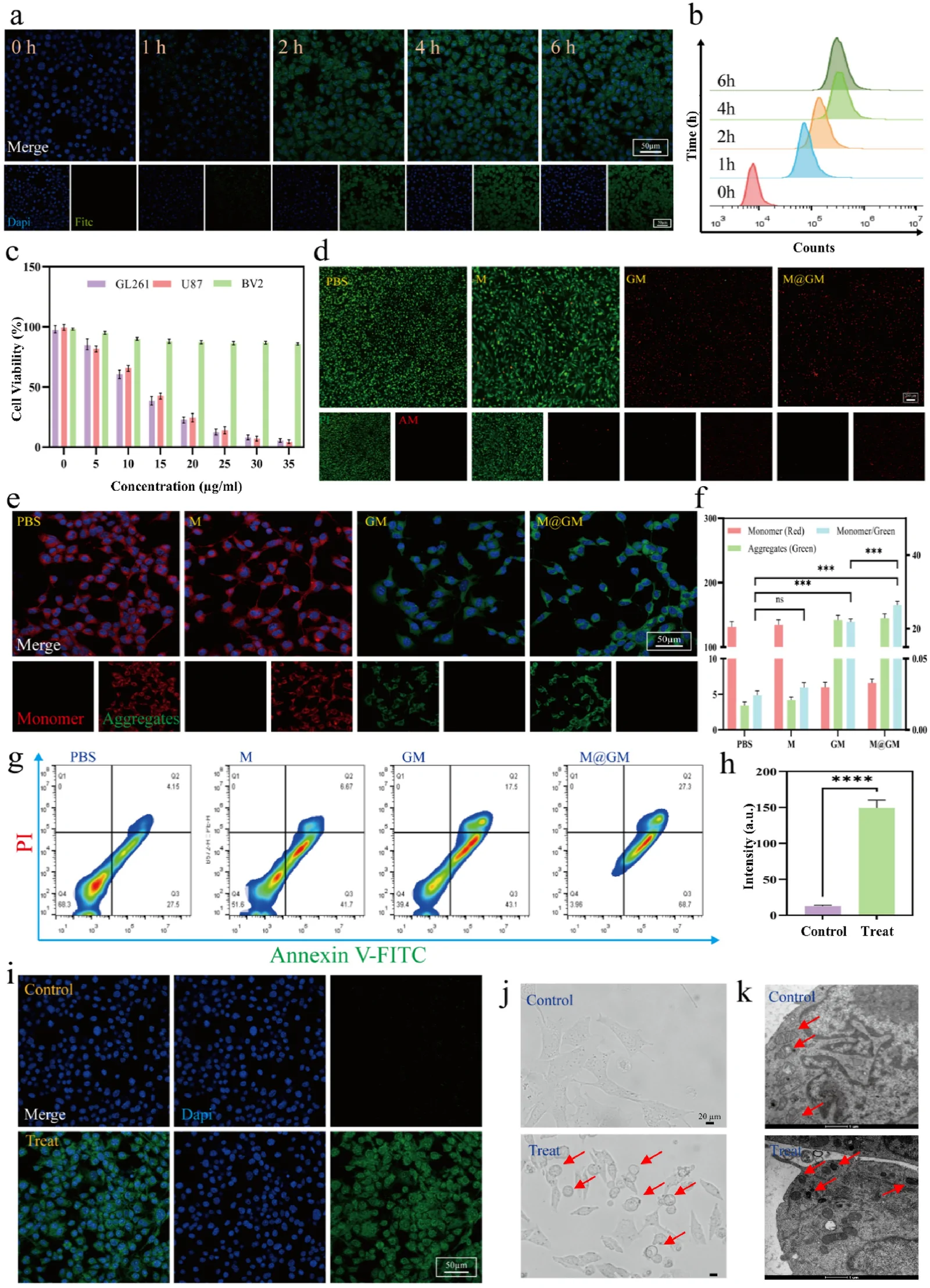
In vitro cellular uptake, cytotoxicity, mitochondrial dysfunction, and cell death induced by the biomimetic M@GM nanosystem. (a) CLSM images showing the cellular uptake of FITC-labeled M@GM by GL261 cells after incubation for 0, 1, 2, 4, and 6 h. (b) Flow cytometric analysis of intracellular fluorescence intensity at different incubation times. (c) Cell viability of GL261, U87, and BV2 cells after treatment with different concentrations of M@GM. (d) Representative live/dead staining images of GL261 cells following different treatments. (e) CLSM images of JC-1 staining after different treatments. (f) Quantitative analysis of JC-1 fluorescence intensity. (g) Flow cytometric analysis of apoptosis after different treatments. (h) Quantitative analysis of intracellular CO fluorescence intensity. (i) CLSM images of intracellular CO fluorescence in GL261 cells after treatment. Scale bar: 50 μm. (j) Representative bright-field images showing morphological changes in GL261 cells after treatment. Scale bar: 20 μm. (k) Representative TEM images showing mitochondrial ultrastructure in GL261 cells before and after treatment. Data are presented as mean ± SD. ns, not significant; ***P < 0.001; ****P < 0.0001.

The biocompatibility of M@GM was subsequently evaluated using the Cell Counting Kit-8 (CCK-8) assay. As shown in Fig. 3c, the viability of GL261 and U87 cells gradually decreased with increasing concentrations of M@GM, whereas negligible cytotoxicity was observed toward BV2 cells within the tested concentration range. In addition, Calcein-AM/propidium iodide (PI) double staining demonstrated that M@GM treatment induced a markedly higher level of cell death than the other treatment groups (Fig. 3d).

### 3.5 In Vitro antitumor mechanism of M@GM

To investigate intracellular CO generation and its association with mitochondrial stress, intracellular CO levels were detected using a CO-specific fluorescent probe. Confocal laser scanning microscopy (CLSM) images showed a markedly enhanced CO fluorescence signal in GL261 cells treated with M@GM compared with the other groups (Fig. 3h and i). The presence of Mn^2+^ may contribute to ROS generation through Fenton-like chemistry. Consistently, the GM and M@GM groups exhibited substantially elevated intracellular ROS levels. Flow cytometric analysis confirmed that the GM and M@GM groups exhibited substantially elevated intracellular ROS levels (Fig. S7).

The effect of M@GM on mitochondrial function was further evaluated by monitoring changes in mitochondrial membrane potential (MMP) using JC-1 staining. CLSM images and quantitative analysis demonstrated that treatment with GM or M@GM significantly increased the green fluorescence of JC-1 monomers while decreasing the red fluorescence of JC-1 aggregates, indicating a pronounced reduction in mitochondrial membrane potential following nanoparticle treatment (Fig. 3e and f). Consistently, transmission electron microscopy (TEM) revealed obvious mitochondrial swelling together with disrupted and dissolved mitochondrial cristae in GL261 cells treated with M@GM (Fig. 3k).

Cell death was further assessed by Annexin V-FITC/PI double staining followed by flow cytometric analysis. As shown in Fig. 3g, the M@GM-treated group exhibited a markedly increased cell-death population. In addition, inverted optical microscopy showed that a proportion of M@GM-treated cells displayed characteristic pyroptotic morphological changes, including plasma membrane swelling and the generation of large bubble-like structures, as indicated by the arrows (Fig. 3j). The concurrent CO release, ROS accumulation, mitochondrial depolarization, and ultrastructural damage suggest that mitochondrial stress represents an important upstream event in M@GM-induced cell death. This mitochondrial injury may subsequently facilitate Caspase-3 activation and downstream GSDME cleavage.

### 3.6 M@GM induces apoptosis/pyroptosis and activates innate immune signaling

To further investigate the molecular mechanisms underlying M@GM-induced cell death, the expression of apoptosis- and pyroptosis-related proteins was evaluated by Western blot analysis. The levels of Caspase-3 and cleaved Caspase-3 (Cle-casp-3) were increased in the M@GM-treated group, with Cle-casp-3 indicating Caspase-3 activation (Fig. 4a–g and S8). The activation of the pyroptotic pathway was subsequently examined. Western blot analysis demonstrated significant alterations in pyroptosis-related proteins following GM and M@GM treatment. Owing to the delivery of exogenous GSDME by the nanoparticles, intracellular GSDME expression was markedly increased and subsequently cleaved into its active N-terminal fragment (GSDME-N) (Fig. 4a–h and i). Consistently, CLSM and quantitative fluorescence analysis showed significantly enhanced green fluorescence of GSDME and red fluorescence of GSDME-N in the M@GM-treated cells (Fig. 4j–l). To further clarify the functional involvement of the Caspase-3/GSDME axis, additional loss-of-function experiments were performed. As shown in Fig. S9 and M@GM treatment markedly increased the levels of Cle-casp-3, GSDME, and GSDME-N compared with PBS. GSDME knockdown substantially attenuated GSDME and GSDME-N accumulation, whereas pharmacological inhibition of Caspase-3 markedly reduced Cle-casp-3 and GSDME-N generation. Notably, simultaneous GSDME knockdown and Caspase-3 inhibition produced the strongest attenuation of these molecular responses. These findings provide additional functional evidence that Caspase-3 activity and GSDME availability are both involved in the pyroptotic response induced by M@GM. Importantly, GSDME-associated signals remained detectable after incorporation into the M@GM formulation, suggesting that the GSDME payload was retained during nanoparticle fabrication and remained available for subsequent proteolytic activation (Fig. S10). Collectively, these findings support a contributory role of the Caspase-3/GSDME axis in the pyroptosis-associated response induced by M@GM.

**Fig. 4:**
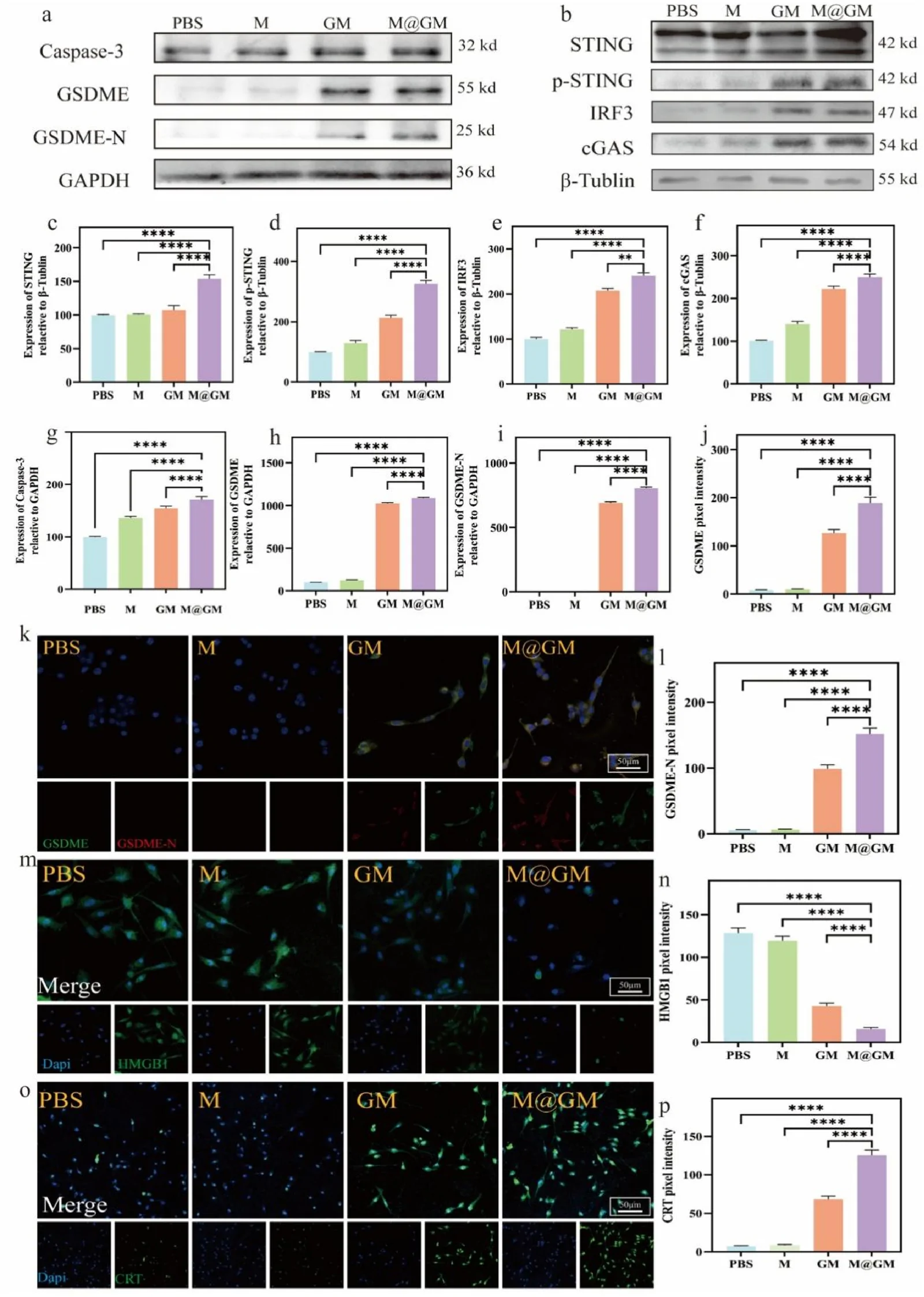
Molecular mechanism underlying M@GM-induced apoptosis/pyroptosis and innate immune activation. (a) Western blot analysis of apoptosis- and pyroptosis-related proteins, including Caspase-3, GSDME, and GSDME-N, in GL261 cells after different treatments. (b) Western blot analysis of cGAS-STING signaling pathway-related proteins, including STING, p-STING, IRF3, cGAS, and β-Tubulin. (c-f) Quantitative analysis of the relative protein expression levels of STING, p-STING, IRF3, and cGAS. (g-i) Quantitative analysis of the relative expression levels of Caspase-3, GSDME, and GSDME-N. (j) Quantification of intracellular GSDME fluorescence intensity. (k) Representative CLSM images of GSDME (green) and GSDME-N (red) expression in GL261 cells following different treatments. (l) Quantitative analysis of GSDME-N fluorescence intensity. (m) Representative immunofluorescence images showing HMGB1 expression in GL261 cells after different treatments. (n) Quantitative analysis of HMGB1 fluorescence intensity. (o) Representative immunofluorescence images showing CRT exposure in GL261 cells after different treatments. (p) Quantitative analysis of CRT fluorescence intensity. Scale bar: 50 μm. Data are presented as mean ± SD. Statistical significance is indicated as ns, *P < 0.05, **P < 0.01, ***P < 0.001, and ****P < 0.0001.

Following mitochondrial disruption, the ability of M@GM to induce immunogenic cell death (ICD) was further evaluated by examining the exposure and release of damage-associated molecular patterns (DAMPs). Immunofluorescence staining demonstrated a pronounced increase in HMGB1 release in M@GM-treated tumor cells compared with the other treatment groups (Fig. 4m and n). In addition, M@GM treatment produced a markedly stronger calreticulin (CRT) fluorescence signal than the control groups (Fig. 4o and p), consistent with an enhanced ICD-associated response. The activation of the cGAS-STING signaling pathway was further assessed. Western blot analysis showed that M@GM treatment significantly upregulated the expression of cGAS-STING pathway-related proteins compared with the control groups (Fig. 4b–f). The loss-of-function experiments further support the functional involvement of the Caspase-3/GSDME axis in M@GM-induced pyroptosis. Importantly, the accompanying HMGB1 release, CRT exposure, and cGAS-STING activation indicate that pyroptotic tumor-cell death may couple direct cytotoxicity with subsequent immune stimulation.

### 3.7 Brain targeting capability and in vivo biosafety evaluation of M@GM

Efficient brain targeting is essential for achieving effective antitumor therapy. Therefore, the brain tumor-targeting capability and systemic biosafety of M@GM were further evaluated in vivo. Whole-body fluorescence imaging performed at 1, 2, 4, and 8 h after intravenous administration (Fig. 5a) showed that M@GM accumulated more rapidly and exhibited stronger fluorescence signals at the intracranial tumor site than the unmodified GM nanoparticles. Ex vivo fluorescence imaging of major organs collected at 8 h further confirmed the persistent brain-associated accumulation of M@GM (Fig. 5b).

**Fig. 5:**
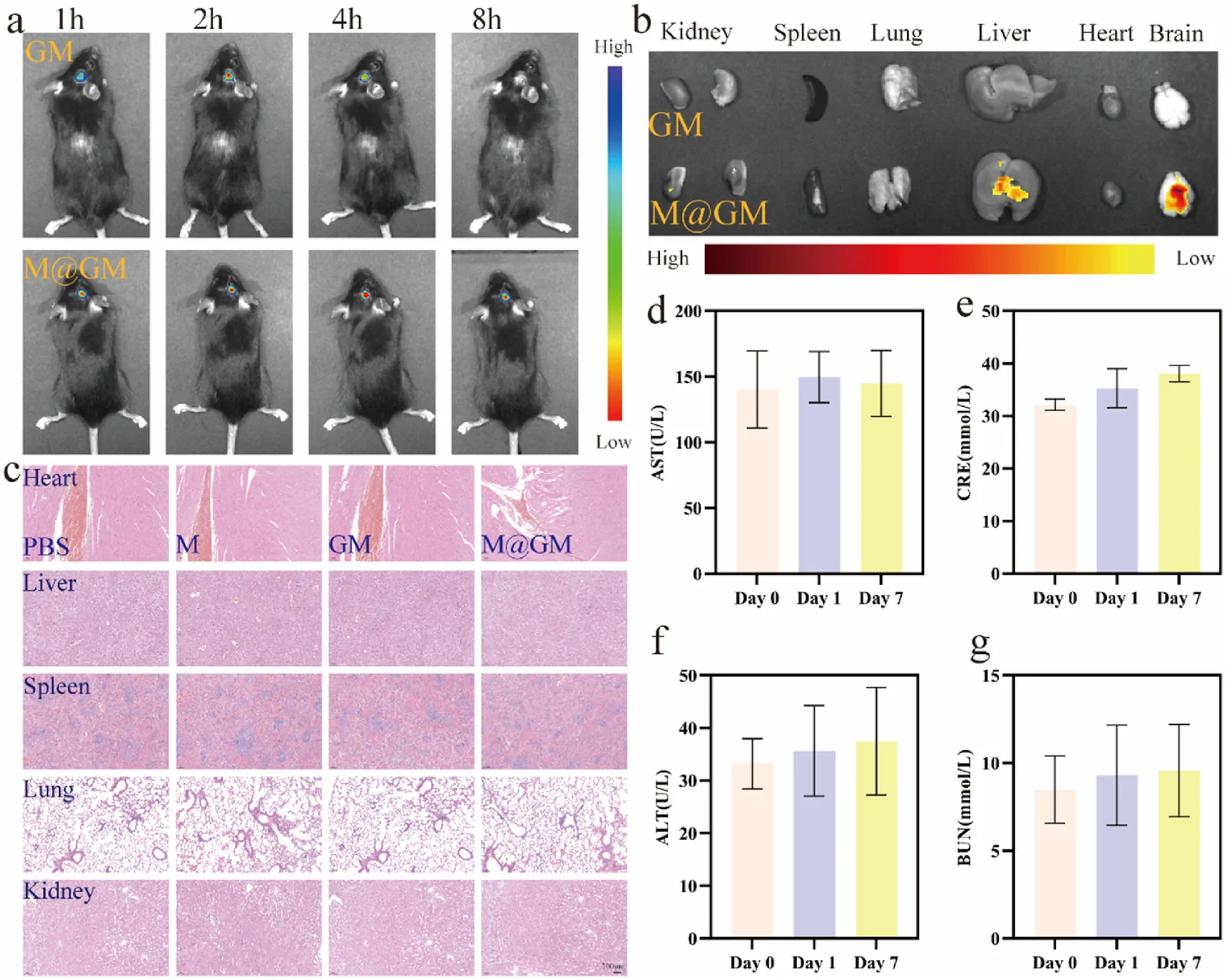
In vivo brain-targeting capability and systemic biosafety evaluation of the biomimetic M@GM nanosystem. (a) Representative in vivo fluorescence images of orthotopic glioma-bearing mice following intravenous administration of GM or M@GM at 1, 2, 4, and 8 h. (b) Ex vivo fluorescence imaging of major organs collected 8 h after intravenous injection of GM or M@GM. (c) Representative H&E staining images of the major organs harvested from mice after different treatments. Scale bar: 100 μm. (d-g) Serum biochemical analyses evaluating systemic biosafety after M@GM administration, including AST, CRE, ALT, and BUN levels measured on days 0, 1, and 7 after treatment. Data are presented as mean ± SD.

Subsequently, the in vivo biosafety profile of M@GM was evaluated. Histological examination of major organs by hematoxylin and eosin (H&E) staining showed no observable signs of tissue damage, including necrosis, inflammatory cell infiltration, or pathological alterations, in mice receiving any of the treatments (Fig. 5c). Serum biochemical analyses were further performed to evaluate hepatic and renal function. As shown in Fig. 5d–g, the levels of alanine aminotransferase (ALT), aspartate aminotransferase (AST), creatinine (CRE), and blood urea nitrogen (BUN) remained within the normal range on days 0, 1, and 7 after administration in the M@GM-treated group, indicating no detectable hepatic or renal toxicity.

### 3.8 In vivo antitumor efficacy of M@GM

Given the ability of M@GM to induce apoptosis/pyroptosis and activate antitumor immunity in vitro, its therapeutic efficacy was further evaluated in an orthotopic GL261 glioblastoma model. The treatment schedule is illustrated in Fig. 6a. GL261 tumor-bearing mice were randomly assigned to 4 groups (PBS, M, GM, and M@GM), and body weight and tumor progression were continuously monitored throughout the treatment period. Fluorescence imaging was performed to evaluate intracranial tumor growth. Compared with the PBS group, mice treated with M@GM exhibited a progressive reduction in intracranial fluorescence intensity, with a pronounced decrease observed on day 9 (Fig. 6b–e).

**Fig. 6:**
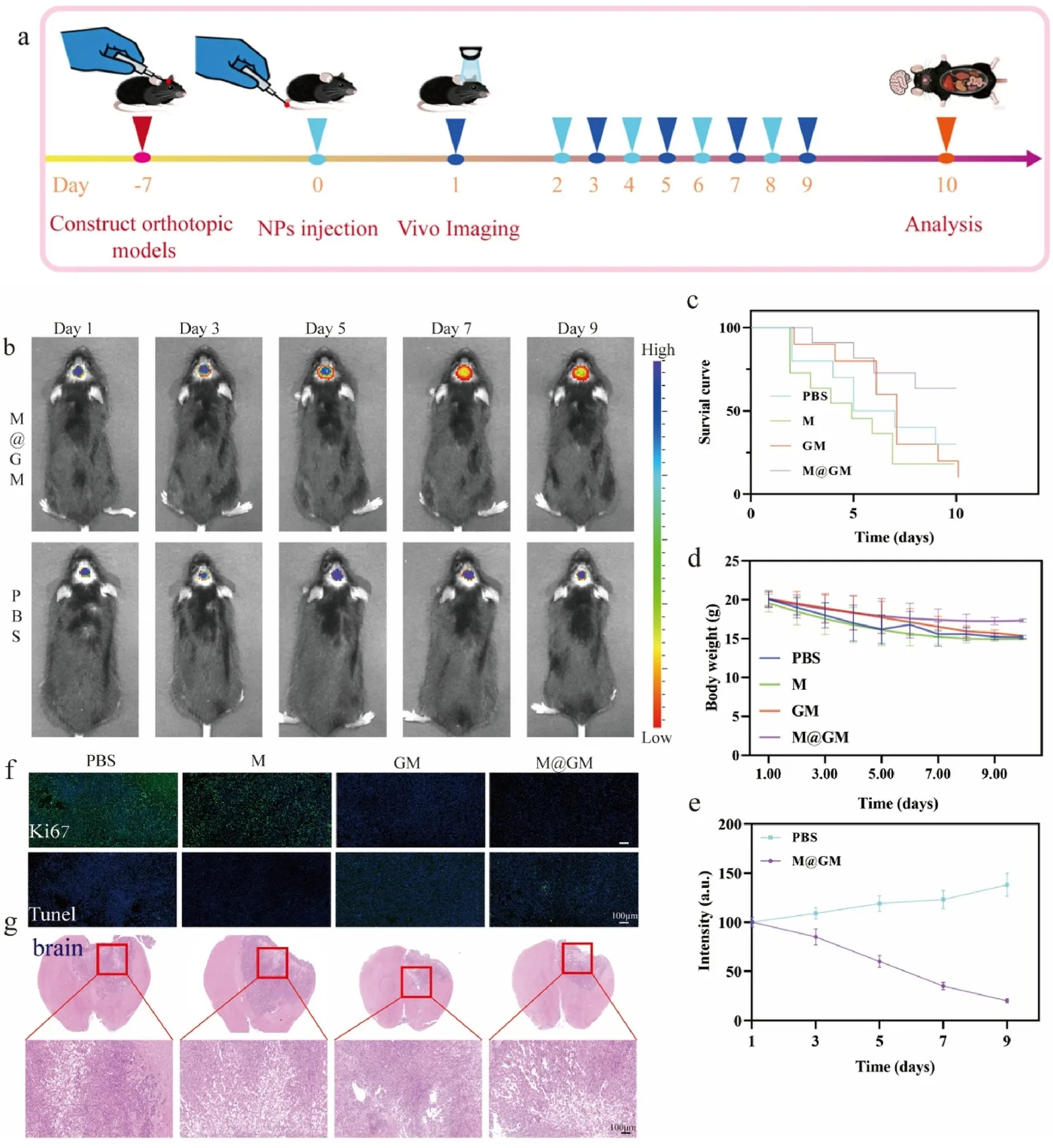
In vivo antitumor efficacy of M@GM in an orthotopic glioblastoma model. (a) Experimental timeline for orthotopic tumor establishment, nanoparticle administration, in vivo imaging, and tissue analysis. (b) Representative in vivo fluorescence images of orthotopic glioma-bearing mice after different treatments. (c) Kaplan-Meier survival analysis. (d) Body weight changes during treatment. (e) Quantification of intracranial fluorescence intensity. (f) Representative Ki67 and TUNEL staining of tumor sections after different treatments. (g) Representative H&E staining of brain sections showing histopathological changes in tumor tissues. Enlarged views of the boxed regions are shown below. Scale bar: 100 μm.

Survival analysis showed that M@GM treatment markedly prolonged the survival of tumor-bearing mice compared with the other treatment groups (Fig. 6c). Body weight monitoring demonstrated a gradual decrease in all groups during treatment; however, the body weight loss was alleviated in the M@GM-treated mice compared with the PBS group (Fig. 6d). To further evaluate the therapeutic effect of M@GM, brain tissues were collected on day 10 after treatment for histological and immunofluorescence analyses. Hematoxylin and eosin (H&E) staining revealed extensive tumor tissue destruction in the M@GM-treated group (Fig. 6g). To further verify the anti-tumor effect, the tumor areas in H&E-stained brain sections were quantitatively analyzed. When normalized to the PBS group (100%), the relative tumor area in the GM group was approximately 50%, while that in the M@GM group was markedly reduced to approximately 30% (Fig. S11). Moreover, M@GM-treated tumor tissues exhibited extensive necrosis, decreased cell density, and blurred tumor boundaries, indicating pronounced tumor destruction. Ki67 immunofluorescence staining showed markedly reduced green fluorescence in the M@GM group compared with the other treatment groups, while TUNEL staining exhibited a substantially stronger fluorescence signal, indicating increased tumor cell death (Fig. 6f). In addition, immunofluorescence staining demonstrated increased expression of Caspase-3, GSDME, and GSDME-N in tumor tissues from the M@GM-treated group (Fig. S12). The enhanced in vivo antitumor efficacy of M@GM may reflect the combined effects of brain-targeted delivery, tumor-responsive activation, mitochondrial stress, and Caspase-3/GSDME-associated pyroptosis.

### 3.9 M@GM remodels the tumor immune microenvironment in vivo

Given the potent therapeutic efficacy of M@GM, its ability to elicit antitumor immune responses in vivo was further investigated. To determine whether M@GM could induce immunogenic cell death (ICD) in tumor tissues, immunofluorescence staining was performed to evaluate the expression of the ICD-associated markers calreticulin (CRT) and high mobility group box 1 (HMGB1). Compared with the other treatment groups, M@GM markedly increased CRT expression and HMGB1 release in tumor tissues (Fig. S13).

The maturation of dendritic cells (DCs) in tumor-draining lymph nodes (TDLNs) was subsequently analyzed by flow cytometry. As shown in Fig. 7a, b and S14, the percentage of mature CD80^+^CD86^+^ DCs increased from 16.90% in the PBS group to 30.89% in the M@GM-treated group. The phenotype of tumor-associated macrophages (TAMs) was further evaluated. Compared with the control group, the proportion of pro-inflammatory M1-type macrophages increased 2.04-fold to 41.56% following M@GM treatment (Fig. 7c, d and S15). In contrast, M2-type macrophages accounted for 89.06% of the macrophage population in the control group, whereas M@GM treatment reduced the proportion of M2-type macrophages to 13.07% (Fig. 7e, f and S16). Tumor-infiltrating T lymphocytes were further analyzed by flow cytometry. As shown in Fig. 7g–j and **S17**, the percentages of CD8^+^ and CD4^+^ T cells increased to 14.07% and 97.19%, respectively, in the M@GM-treated group. In addition, regulatory T cells (Tregs) were evaluated, and the results showed a reduced proportion of Tregs following M@GM treatment (Fig. 7k, l and S18).

**Fig. 7:**
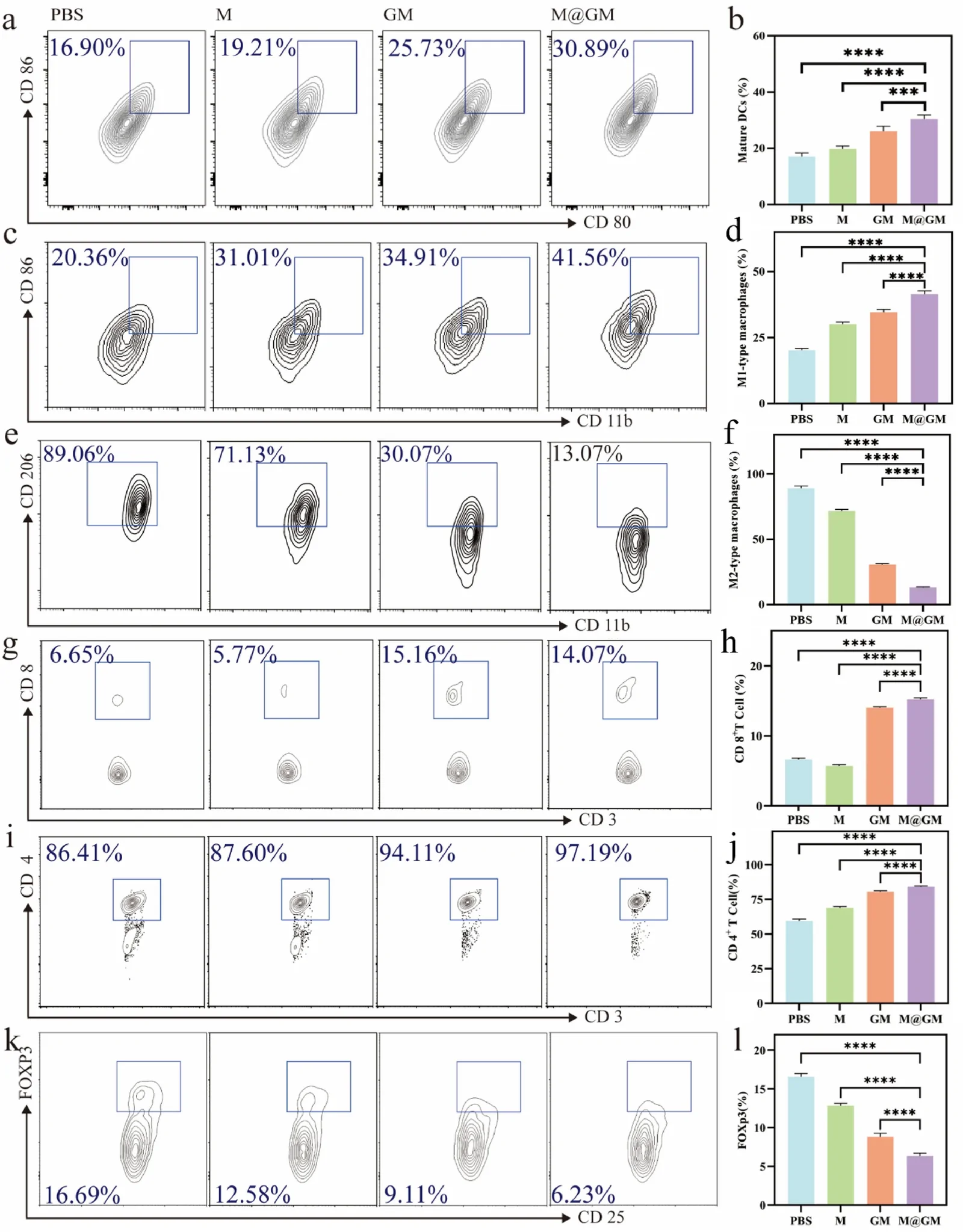
In vivo immune remodeling induced by M@GM in orthotopic glioblastoma-bearing mice. (a, b) Flow cytometric analysis and quantification of mature dendritic cells (CD80^+^CD86^+^) in TDLNs. (c, d) Representative flow cytometry plots and quantitative analysis of M1 macrophages in tumor tissues. (e, f) Representative flow cytometry plots and quantitative analysis of M2 macrophages. (g, h) Representative flow cytometry plots and quantitative analysis of tumor-infiltrating CD8^+^ T cells. (i, j) Representative flow cytometry plots and quantitative analysis of tumor-infiltrating CD4^+^ T cells. (k, l) Representative flow cytometry plots and quantitative analysis of regulatory T cells (Tregs). Data are presented as mean ± SD. ***P < 0.001, and ****P < 0.0001.

To further evaluate the functional activation of infiltrating CD8^+^ T cells, intracellular IFN-γ and Granzyme B expression was analyzed by flow cytometry. The proportion of IFN-γ^+^CD8^+^ T cells increased from 27.4% in the PBS group to 53.1% in the M@GM group, while Granzyme B^+^CD8^+^ T cells increased from 34.6% to 66.4% (Fig. S19 and S20). These findings indicate that M@GM effectively enhanced the functional activation of cytotoxic T cells in the tumor microenvironment. Together with DC maturation and M1 polarization, these findings suggest coordinated remodeling of the immunosuppressive GBM microenvironment.

### 3.10 Transcriptomic analysis

To characterize treatment-associated transcriptional changes underlying the antitumor effects of M@GM against GL261 tumors, transcriptomic sequencing was performed on brain tissues collected from the M@GM-treated and control groups. Under the conditions that P-value < 0.05 and |log2FC| ≥ 1.0. Compared with the control group, a total of 3100 differentially expressed genes (DEGs) were identified in the M@GM-treated group, including 1613 upregulated and 1487 downregulated genes (Fig. 8a). The identified DEGs included genes associated with apoptosis (Bax, Cytc), pyroptosis (Gsdme), and proliferation-related signaling (Egfr). Gene Ontology (GO) enrichment analysis was subsequently conducted to characterize the biological functions of the identified DEGs (Fig. 8b). In the Biological Process (BP) category, DEGs were predominantly enriched in immune system processes, response to stimulus, regulation of cellular processes, and multicellular organismal processes, indicating extensive alterations in immune-related biological activities. In the Cellular Component (CC) category, significantly enriched terms included the extracellular region, membrane-associated structures, and organelle components. Within the Molecular Function (MF) category, binding activity, catalytic activity, and molecular function regulator were the most significantly enriched terms, reflecting broad changes in intracellular molecular signaling networks. Kyoto Encyclopedia of Genes and Genomes (KEGG) pathway enrichment analysis was further performed to identify the major signaling pathways associated with the therapeutic response (Fig. 8c). The DEGs were significantly enriched in multiple cell death- and inflammation-related pathways, including necroptosis and apoptosis. A chord diagram further illustrated the interaction network between the enriched pathways and the associated DEGs (Fig. 8d), showing the associations among apoptosis-, necroptosis-, and immune-related pathways and their corresponding DEGs.

**Fig. 8:**
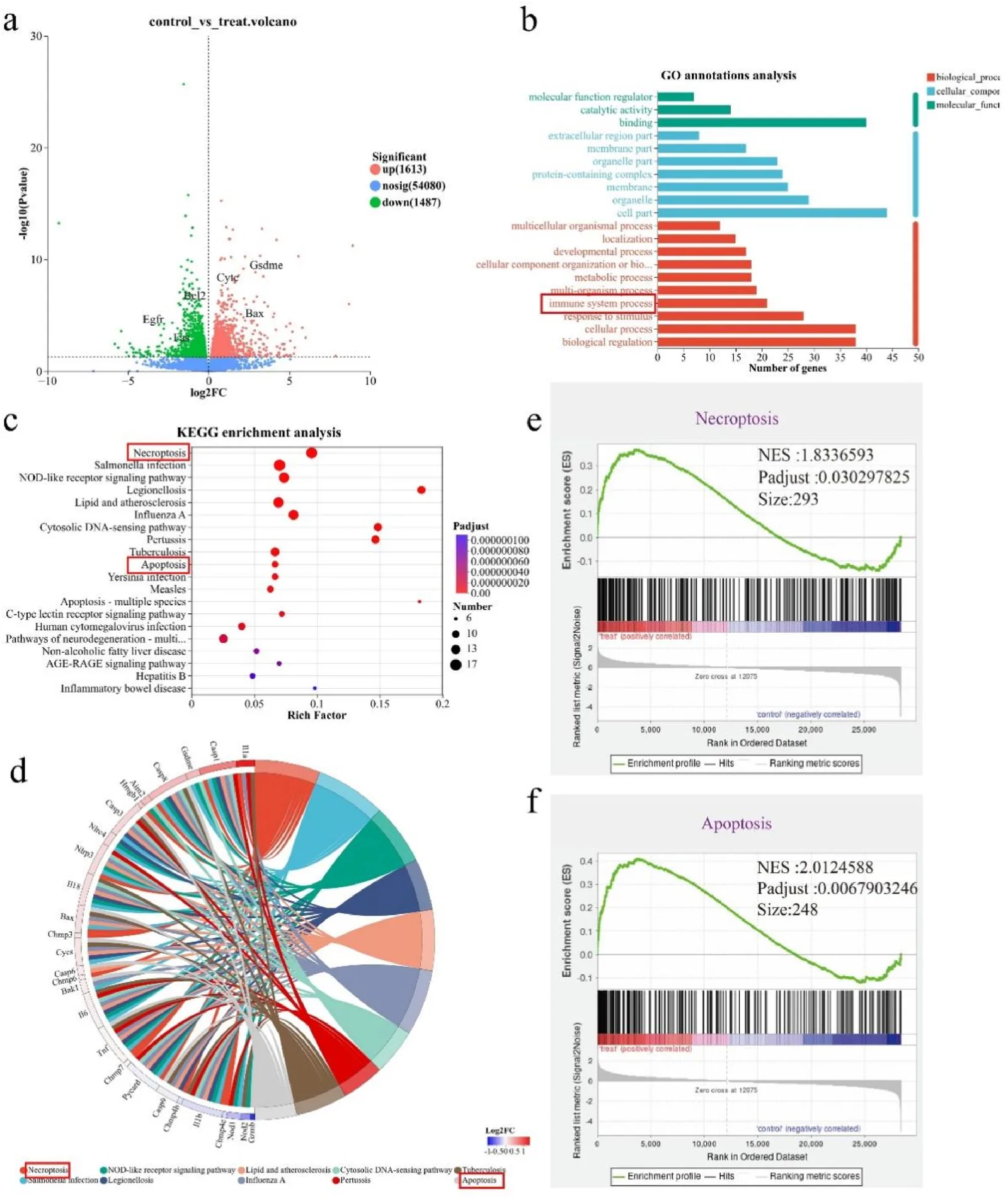
Transcriptomic profiling of tumor tissues following M@GM treatment. (a) Volcano plot showing DEGs between the indicated groups (n = 3). (b) GO enrichment analysis of DEGs. (c) KEGG pathway enrichment analysis of DEGs. (d) Chord diagram illustrating the associations between representative enriched pathways and corresponding differentially expressed genes. (e) GSEA demonstrating significant enrichment of the necroptosis-related gene signature following M@GM treatment. (f) GSEA showing significant enrichment of the apoptosis-related gene set.

To further assess pathway-associated gene-set enrichment of these pathways at the gene-set level, gene set enrichment analysis (GSEA) was performed (Fig. 8e and f). The necroptosis gene set exhibited significant positive enrichment with a normalized enrichment score (NES) of 1.834 and an adjusted P value of 0.0303. Similarly, the apoptosis gene set showed significant positive enrichment, with an NES of 2.012 and an adjusted P value of 0.0068. These results indicate positive enrichment of apoptosis- and necroptosis-related gene sets following M@GM treatment.

## 4 Conclusions

In summary, biomimetic microglia membrane-coated nanoplatform (M@GM) was successfully constructed for glioblastoma therapy by integrating brain-targeted delivery, mitochondrial disruption, pyroptosis induction, and immune activation. Benefiting from microglial membrane camouflage, M@GM efficiently crossed the blood-brain barrier and preferentially accumulated in intracranial tumors. Upon tumor-responsive degradation, the released CO and Mn^2+^ synergistically induced mitochondrial dysfunction and oxidative stress, accompanied by activation of the Caspase-3/GSDME axis and enhanced pyroptosis-associated cell death. M@GM treatment was associated with enhanced cGAS-STING signaling, accompanied by dendritic cell maturation, macrophage polarization, and increased CD4^+^/CD8^+^ T-cell infiltration while reducing immunosuppressive Treg cells, thereby remodeling the glioblastoma immune microenvironment. These biological effects translated into effective inhibition of orthotopic glioblastoma growth, prolonged survival, and favorable biosafety in vivo. Overall, this study presents a biomimetic cascade therapeutic strategy based on brain-targeted delivery, mitochondrial disruption, pyroptosis-associated responses, and immune remodeling, providing a promising approach for glioblastoma nanotherapy and immunotherapy.

## CRediT authorship contribution statement

**Guangyu Ma:** Formal analysis, Data curation. **Junjie Li:** Methodology, Investigation, Funding acquisition. **Kai Wang:** Formal analysis, Data curation. **Dehong Yu:** Formal analysis, Data curation. **Jiale Li:** Formal analysis, Data curation. **Ming Guan:** Supervision, Resources, Methodology. **Fenglei Gao:** Investigation, Funding acquisition, Formal analysis. **Zhao Liu:** Validation, Supervision, Software, Resources. **Jun Lan:** Validation, Supervision, Software.

## Declaration of competing interest

The authors declare that they have no known competing financial interests or personal relationships that could have appeared to influence the work reported in this paper.

## Data Availability

Data will be made available on request.

## Appendix A Supplementary data

The Supplementary data contain: Instruments, Intracellular ROS Production, Western Blot Analysis, Immunofluorescence Staining, Mitochondrial Integrity Analysis, Intracellular CO Production, Morphological Analysis of Cell Pyroptosis, Evaluation of In Vitro BBB Penetration Ability, Wound Healing Assay, Transwell Cell Migration/Invasion Assay, Orthotopic Glioblastoma Mouse Model, In Vivo Biodistribution Assay in GBM Mouse Model, Immune System Activation in Mice, Anti-Tumor Effect of Nanomaterials, Immunofluorescence Staining of Mouse Brain Tissue.
