# Supplementary material for: A biomimetic gas-protein co-delivery nanoplatform targets mitochondrial stress and GSDME activation for glioblastoma immunotherapy
Source: Mater Today Bio. 2026 Oct 12;41:103750. doi: 10.1016/j.mtbio.2026.103750 (PMC13694755; doi:10.1016/j.mtbio.2026.103750)
Supplement: Supplementary file 1 [file mmc1.doc]

**Supporting Information**

**A Biomimetic Gas-Protein Co-delivery Nanoplatform Targets Mitochondrial Stress and GSDME Activation for Glioblastoma Immunotherapy**

*Guangyu Ma^a,b,c,1^,* *Junjie Li^a,b,c,1^, Kai Wang^f,1^, Dehong Yu^d,1^, Jiale Li^a,b,c^, Ming Guan^a,^*, Fenglei Gao^c,^*, Zhao Liu^b,^* and Jun Lan^e,^**

a. Department of Laboratory Medicine, Huashan Hospital, Shanghai Medical College, Fudan University, Shanghai, 200040, China

b. Department of Thyroid and Breast Surgery, The Affiliated Hospital of Xuzhou Medical University, Xuzhou, Jiangsu 221002, China

c. School of Pharmacy, Xuzhou Medical University, Xuzhou, Jiangsu, 221004, China

d. The Affiliated Pizhou Hospital of Xuzhou Medical University, Jiangsu, 221399, China

e. Department of Orthopaedic Surgery, Lishui Central Hospital and Fifth Affiliated Hospital of Wenzhou Medical University, Zhejiang, 323000, China

f. Department of Neurosurgery, The Affiliated Huai'an Hospital of Xuzhou Medical University, The Second People's Hospital of Huai'an, Huai'an, 223002, China

Corresponding authors. E-mail addresses: guanming88@yahoo.com (M. Guan), xylzhao9999@163.com (Z. Liu), flgao@xzhmu.edu.cn (F. Gao), [atomacro@163.com](mailto:atomacro@163.com) , (J. Lan).

^1^ These authors contributed equally to this research work.

**Contents**

Instruments	**S4**

Reactive Oxygen Species (ROS) Production.	**S4**

**Western Blot Analysis.	S4**

**Immunofluorescence Staining Assay	S5**

**Mitochondrial Integrity Analysis.	S6**

**Verification of Intracellular Carbon Monoxide (CO) Production	S7**

**Morphological Analysis of Cell Pyroptosis	S7**

**Evaluation of In Vitro Blood-Brain Barrier (BBB) Penetration Ability	S7**

**Wound Healing Assay.	S8**

**Morphological Analysis of Cell Pyroptosis	S8**

**Wound Healing Assay	S8**

**Transwell Cell Migration/Invasion Assay (Crystal Violet Staining)	S8**

**Establishment of Orthotopic Glioblastoma Mouse Model	S9**

**In Vivo Biodistribution Assay in GBM Mouse Model	S10**

**Detection of Immune System Activation in Mice	S11**

**Detection of Gene Expression Changes in Mouse Brain	S12**

**Evaluation of the Anti-Tumor Effect of Nanomaterials	S12**

**Immunofluorescence Staining of Mouse Brain Tissue	S13**

**Hematoxylin and Eosin (H&E) Staining of Mouse Tissues	S14**

**Figure S1	S15**

**Figure S2	S15**

**Figure S3	S15**

**Figure S4	S16**

**Figure S5	S16**

**Figure S6	S17**

**Figure S7	S17**

**Figure S8	S17**

**Figure S9	S18**

**Figure S10	S18**

**Figure S11	S18**

**Figure S12	S19**

**Figure S13	S19**

**Figure S14	S19**

**Figure S15	S19**

**Figure S16	S20**

**Figure S17	S20**

**Figure S18	S20**

**Figure S19	S20**

**Figure S20	S21**

Experimental Section

Instruments. Nanoparticles were obtained by centrifugation using a desktop centrifuge (H/T16MM, Herexi, China). The morphology of the samples was observed under the TecnaiTM G2 TWIN (FEI, USA) transmission electron microscope. Malvern Zetasizer Nano ZS90 was used to measure the zeta potential and hydrodynamic diameter of various samples. A UV1601 spectrophotometer was employed to obtain the UV-Vis-NIR absorbance spectra of the samples. The Bio Rad680 ELISA reader is used for the standard determination of CCK-8. Fluorescence images were obtained on a fluorescence inverted microscope (IX73P1F, Olympus, Japan) and a confocal laser scanning microscope (Leica STELLARIS 5, Germany). Perform flow cytometry analysis on the FACS Canto II flow cytometry (Becton Dickinson, USA). The IVIS imaging system (Perkin Elmer Lumina S5, USA) is used for in vivo fluorescence imaging.

Determination of Intracellular ROS Production. Intracellular ROS levels were detected using the DCFH-DA fluorescent probe. GL261 cells were treated with M@GM for 2 h, the supernatant was discarded, and the cells were washed with PBS. DCFH-DA working solution was added, and the cells were incubated at 37 ℃ in the dark for 45 min. The fluorescence signal was observed under an inverted fluorescence microscope.

**Western Blot Analysis.** Sample preparation: Treated cells were collected and lysed in RIPA lysis buffer containing protease and phosphatase inhibitors on ice for 30 min. The lysate was centrifuged at 12000 rpm for 15 min at 4 ℃, and the supernatant was collected as total protein lysate. The protein concentration was determined by BCA assay. 5×SDS loading buffer was added to the lysate, and the protein was denatured by heating at 95℃ for 5-10 min. SDS-PAGE electrophoresis: Separating gel and stacking gel with appropriate concentrations were prepared according to the molecular weight of target proteins. Equal amounts of denatured protein samples were loaded into the wells. Electrophoresis was performed at a constant voltage of 80 V in the stacking gel, and adjusted to 120 V after the bromophenol blue entered the separating gel, until the indicator migrated to the bottom of the gel. Electrotransfer: The polyvinylidene fluoride (PVDF) membrane was activated with methanol for 1 min. The transfer sandwich was assembled in the order of “filter paper-gel-membrane-filter paper”, and the electrotransfer was performed at a constant voltage of 100 V for 60-90 min under low temperature. Antibody incubation: After electrotransfer, the membrane was blocked with 5% non-fat milk at room temperature for 1 h, then incubated with primary antibody dilution at 4 ℃ overnight with shaking. The membrane was washed 3 times with TBST for 10 min each time, followed by incubation with HRP-conjugated secondary antibody dilution at room temperature for 1 h with shaking, and washed again 3 times with TBST. Development and quantification: The membrane was incubated with ECL chemiluminescence reagent for 1-2 min, and images were acquired by a chemiluminescence imaging system. The gray value of the bands was analyzed by ImageJ software, and the relative expression level of the target protein was normalized to the internal reference protein.

**Immunofluorescence Staining Assay.** Cells were seeded in confocal dishes and cultured until 70%-80% confluence, followed by drug treatment. After incubation, the medium was discarded, and the cells were washed 2-3 times with pre-warmed PBS. The cells were fixed with 4% PFA at room temperature for 15-20 min, washed 3 times with PBS, permeabilized with 0.1%-0.5% Triton X-100 at room temperature for 10-15 min, and washed again 3 times with PBS. The cells were blocked with blocking buffer containing 5% BSA/goat serum at room temperature for 30-60 min. The blocking buffer was discarded, and primary antibody dilution was added, followed by incubation in a wet chamber at 4 ℃ overnight. After washing 3 times with PBS, fluorescence-labeled secondary antibody was added, and the cells were incubated at room temperature in the dark for 1 h. The cells were stained with DAPI working solution at room temperature in the dark for 5 min, washed with PBS, and mounted with anti-fluorescence quenching mounting medium. Images were acquired by CLSM, and the fluorescence intensity was quantitatively analyzed by ImageJ software.

**Mitochondrial Integrity Analysis.** Mitochondrial membrane potential (MMP) was detected using the JC-1 fluorescent probe. GL261 cells were seeded in confocal dishes and cultured until 70%-80% confluence, followed by drug treatment. After incubation, the medium was discarded, and the cells were gently washed twice with pre-warmed PBS. JC-1 staining working solution was added, and the cells were incubated at 37 ℃ in a 5% CO₂ incubator in the dark for 20-30 min. After washing to remove free dye, images of red and green fluorescence channels were acquired by CLSM. The ratio of red to green fluorescence intensity was quantitatively analyzed by ImageJ software to evaluate the mitochondrial membrane potential and damage degree.

**Verification of Intracellular Carbon Monoxide (CO) Production.** Intracellular CO production was detected using the CO-specific fluorescent probe BioTracker Carbon Monoxide dye (SCT051) combined with CLSM. GL261 cells were seeded in confocal dishes and cultured until 70%-80% confluence, then divided into control group and M@GM treatment group. After incubation, the cells were washed twice with pre-warmed PBS, SCT051, PdCl₂ and matched working solution was added, followed by incubation at 37 ℃ in a 5% CO₂ incubator in the dark for 45 min. After nuclear staining with DAPI, images were acquired by CLSM at excitation 450-480 nm and emission 525 nm. The average green fluorescence intensity was quantitatively analyzed by ImageJ software to reflect the intracellular CO production level.

**Morphological Analysis of Cell Pyroptosis.** GL261cells were seeded in 6-well plates at 1×10⁵ cells/well and cultured until 60%-70% confluence, followed by drug treatment. After incubation, the cells were gently washed once with pre-warmed PBS. The cell morphology was observed under an inverted optical microscope with a 10× objective lens, and bright-field images were taken from at least 5 random fields per group to record typical pyroptotic morphological features, including cell swelling, membrane blebbing, and pyroptotic body formation.

**Evaluation of In Vitro Blood-Brain Barrier (BBB) Penetration Ability.** An in vitro BBB model was established using Transwell plates. bEnd.3 brain microvascular endothelial cells were seeded in the upper chamber of Transwell inserts (0.4 μm pore size) at 2×10⁵ cells/well and cultured for 7-10 days until the barrier was mature. The transendothelial electrical resistance (TEER) was measured daily, and the model was considered successfully constructed when TEER > 140 Ω·cm². The medium in the upper chamber was discarded, and the inserts were washed with pre-warmed PBS. Serum-free medium containing M@GM was added to the upper chamber, and 600 μL of serum-free medium was added to the lower chamber. Medium samples were collected from the lower chamber at preset time points. The concentration of M@GM in the lower chamber was quantitatively detected by fluorescence method, and the apparent permeability coefficient (Papp) was calculated to evaluate the BBB penetration ability of M@GM.

**Wound Healing Assay.**GL261 cells were seeded in 6-well plates at 2×10⁵ cells/well and cultured until 90%-100% confluence to form a dense monolayer. A uniform linear scratch was made on the cell monolayer with a sterile 200 μL pipette tip, and the detached cells were removed by gentle washing twice with pre-warmed PBS. The cells were divided into control group and M@GM treatment group, and cultured in medium containing 1% FBS. Bright-field images of the same scratch position were taken under an inverted microscope at 0 h and 24 h, respectively. The scratch width was measured by ImageJ software, and the scratch healing rate was calculated to evaluate cell migration ability.

**Transwell Cell Migration/Invasion Assay.** Transwell inserts (8 μm pore size) were placed in 24-well plates, and 600 μL of complete medium containing 10% FBS was added to the lower chamber. GL261 cells from each treatment group were collected, resuspended in serum-free medium, and seeded in the upper chamber at 1×10⁵ cells/insert. The plates were incubated in a 37 ℃, 5% CO₂ incubator for 24 h. After incubation, the inserts were washed twice gently with PBS, and the non-migrated cells on the upper surface of the membrane were gently removed with a cotton swab. The cells on the lower surface of the membrane were fixed with 4% PFA at room temperature for 15 min, washed with PBS, and stained with 0.1% crystal violet staining solution at room temperature for 10-15 min. Excess dye was rinsed off with clean water. Images were taken from at least 5 random fields under an inverted optical microscope. The number of stained cells was counted by ImageJ software to quantitatively evaluate cell migration/invasion ability.

**Establishment of Orthotopic Glioblastoma Mouse Model.** Cell suspension preparation: RFP-labeled GL261 cells in the logarithmic growth phase were collected, digested with trypsin, and washed twice with pre-cooled PBS. Cell viability was confirmed to be >90% by trypan blue staining. The cell concentration was adjusted to 5×10⁴ cells/μL with pre-cooled PBS, mixed with Matrigel matrix gel at a volume ratio of 1:1, and stored on ice until use. Stereotaxic intracranial inoculation: Mice were anesthetized with isoflurane inhalation and fixed on a brain stereotaxic apparatus to ensure the skull was horizontal. The head was shaved and disinfected alternately with iodophor and 75% ethanol for 3 times. A longitudinal midline incision of about 1.0 cm was made on the scalp to expose the skull. With Bregma as the origin, the target coordinates were determined as: AP + 0.5 mm, ML + 2.0 mm, DV 3.0 mm. A cranial burr hole was drilled at the target site with a dental drill. A 10 μL micro syringe was fixed on the injection arm of the stereotaxic apparatus, and the needle tip was inserted to the target depth. After staying for 1 min, 5 μL of cell suspension (total cell number: 2.5×10⁵) was injected at a speed of 0.5 μL/min. After injection, the needle was left in place for 10 min and then withdrawn slowly. The scalp was sutured and disinfected again. Postoperative care and model monitoring: After surgery, the mice were resuscitated on a 37 ℃ constant temperature pad and housed in single cages. The mental state and incision healing of the mice were observed daily for 3 consecutive days. Intracranial tumor growth was monitored by in vivo small animal fluorescence imaging system at 7 d, 10 d, 14 d, 17 d, and 21 d after modeling. Body weight and neurobehavioral changes of the mice were monitored daily. Humane endpoint: Mice were immediately euthanized when any of the following conditions occurred: a. body weight decreased by >20% from baseline and failed to recover for 2 consecutive days; b. severe neurological dysfunction; c. moribund state.

**In Vivo Biodistribution Assay in GBM Mouse Model.** 20 GBM model mice were randomly divided into 4 groups (n = 5 per group), which were injected with 100 μL of PBS, 50 μg/mL FITC-M, 50 μg/mL FITC-GM, and 50 μg/mL FITC-M@GM via the tail vein, respectively. Whole-body fluorescence images were acquired by the in vivo imaging system at 1 h, 2 h, 4 h, and 8 h after administration under isoflurane anesthesia to analyze the in vivo distribution and brain enrichment of the formulations. After the last imaging at 8 h post-administration, the mice were euthanized. The heart, liver, spleen, lung, kidney, and brain tissue were harvested, rinsed with pre-cooled sterile PBS, blotted dry with filter paper, and imaged ex vivo by the in vivo imaging system for quantitative analysis of fluorescence distribution in each organ.

**Detection of Immune System Activation in Mice.** Immune cell isolation: At the experimental endpoint, the mice were euthanized, and the spleen and tumor tissue were harvested under sterile conditions and placed in pre-cooled PBS containing 2% FBS. The spleen was ground and filtered through a 70 μm cell strainer. The tumor tissue was minced and digested with RPMI 1640 medium containing 1 mg/mL collagenase Ⅳ and 0.1 mg/mL DNase Ⅰ at 37 ℃ with shaking for 30-60 min, followed by filtration through a 70 μm cell strainer. The cell suspension was centrifuged at 300×g for 5 min at 4 ℃. Red blood cells were lysed with 1× red blood cell lysis buffer at room temperature for 3-5 min. The lysis was terminated, and the cells were centrifuged again. Viable cells were counted by trypan blue staining, and the concentration was adjusted to 1×10⁷ cells/mL for subsequent use. Flow cytometric surface staining: 1×10⁶ cells per sample were resuspended in 100 μL PBS, blocked with 10% goat serum at 4 ℃ in the dark for 30 min. The cells were centrifuged at 300×g for 5 min at 4 ℃, and the supernatant was discarded. Fluorescence-labeled surface antibody cocktail was added, followed by incubation at 4 ℃ in the dark for 30 min. The cells were washed 3 times with FACS buffer and transferred to flow tubes for on-machine detection. Intranuclear protein staining: After surface staining and washing, the cells were resuspended in permeabilization working solution and incubated at 4 ℃ in the dark for 30-60 min. The cells were washed twice with permeabilization washing buffer, and fluorescence-labeled intracellular antibody was added, followed by incubation at 4 ℃ in the dark for 30-60 min. After washing again, the cells were resuspended in FACS buffer and detected by flow cytometry to analyze the proportion of immune cell subsets.

**Detection of Gene Expression Changes in Mouse Brain Before and After Treatment.** At the experimental endpoint, the mice were euthanized, and the brain tissue was quickly dissected on ice. Blood vessels and connective tissue were removed, and the tissue was rinsed with RNase-free PBS, cut into pieces ≤5 mm³, blotted dry with sterile filter paper, and snap-frozen in liquid nitrogen for more than 10 min. The samples were stored in a -80 ℃ ultra-low temperature freezer for subsequent transcriptome sequencing to analyze differentially expressed genes and pathway enrichment before and after treatment.

**Evaluation of the Anti-Tumor Effect of Nanomaterials on Orthotopic Tumors in Mice.** Experimental grouping and administration: After successful establishment of the orthotopic glioma model, the mice were randomly divided into PBS control group and M@GM treatment group, with 10 mice in each group. Starting from Day 0 (the first day after successful modeling), the mice were administered once daily via tail vein injection. The M@GM group was given a dose of 10 mg/kg, and the control group was injected with an equal volume of sterile PBS. Tumor growth monitoring: Intracranial tumor growth was detected by in vivo bioluminescence imaging every 2 days starting from Day 0. The body weight of the mice was weighed at a fixed time daily, and the mental state, food intake, and activity of the mice were recorded. Survival evaluation: The survival time of mice in each group was recorded, and the Kaplan-Meier survival curve was drawn to analyze the effect of M@GM on the survival period of tumor-bearing mice.

**Immunofluorescence Staining of Mouse Brain Tissue.** Sample preparation: At the experimental endpoint, the mice were euthanized, and the brain tissue was quickly isolated and fixed in 4% PFA at 4 ℃ for 24 h. The tissue was dehydrated in 20% and 30% sucrose solutions at 4 ℃ successively until it sank to the bottom. The brain tissue was embedded in OCT embedding medium and snap-frozen in liquid nitrogen. Coronal sections with a thickness of 10 μm were cut with a freezing microtome, attached to poly-L-lysine-coated adhesive slides, and stored at -80℃ for subsequent use. Staining procedure: The sections were equilibrated at room temperature for 30 min, permeabilized with 0.3% Triton X-100 at room temperature for 10 min, and rinsed 3 times with PBS. The sections were blocked with blocking buffer containing 10% goat serum at room temperature for 1 h. The blocking buffer was discarded, and primary antibody dilution was added dropwise, followed by incubation in a wet chamber at 4℃ overnight. After rinsing 3 times with PBS, the corresponding fluorescence secondary antibody was added dropwise, and the sections were incubated at room temperature in the dark for 1 h. After rinsing with PBS, the sections were stained with DAPI working solution at room temperature in the dark for 5 min, and mounted with anti-fluorescence quenching mounting medium. Imaging and analysis: Images were acquired by CLSM with unified imaging parameters. The fluorescence expression level of target proteins was quantitatively analyzed by ImageJ software.

**Hematoxylin and Eosin (H&E) Staining of Mouse Tissues.** Sample preparation: At the experimental endpoint, the mice were euthanized, and the heart, liver, spleen, lung, and brain tissue were harvested and fixed in 4% PFA at 4 ℃ for 24 h. The tissues were dehydrated in gradient ethanol, cleared in xylene, infiltrated with paraffin, and embedded in paraffin blocks. Serial sections with a thickness of 5 μm were cut with a paraffin microtome, spread in a 45 ℃-water bath, attached to glass slides, and baked at 60 ℃ for 2 h. Staining procedure: The sections were dewaxed in xylene, rehydrated in gradient ethanol to distilled water, stained with hematoxylin solution for 5 min, rinsed with running water for blueing, differentiated with 1% hydrochloric acid alcohol, and rinsed with running water for 10 min. The sections were then stained with eosin solution for 2 min, dehydrated in gradient ethanol, cleared in xylene, and mounted with neutral balsam. Microscopic examination: The tissue structure, cell morphology, and inflammatory infiltration of the main organs were observed under an optical microscope to evaluate the in vivo systemic safety of the nanomaterials. Meanwhile, the cell density, necrosis degree, and tumor boundary of the brain tumor area were observed to comprehensively evaluate the in vivo anti-tumor effect of M@GM.

**Figure S1.** Scanning electron microscopy (SEM) image of M@GM nanoparticles, showing spherical morphology and monodisperse distribution. Scale bar = 200 nm.

**Figure S2.** (a) UV-vis calibration curve of GSDME at different concentrations. (b) UV-vis calibration curve of MnCO at different concentrations. (c) UV-vis absorption spectra of the supernatant collected after M@GM preparation.

**Figure S3.** (a) In vitro cumulative release of CO from M@GM at different pH values with or without addition of H_2_O_2_. (b) The release of Mn^2+^ from M@GM in PBS solutions (pH 7.4, 6.5 and 5.4) with or without addition of H_2_O_2_.

**Figure S4**. In vitro blood-brain barrier (BBB) penetration assay using a Transwell model. Total fluorescence intensity in the lower chamber after incubation with GM or M@GM for 0.5 h or 2 h. Data are presented as mean ± SD (n = 3). ns, not significant; ***P < 0.001, ****P < 0.0001.

**Figure S5**. Fluorescence images show the penetration of M@GM across the in vitro BBB at 0.5 h and 2 h with or without anti-VCAM-1.

**Figure S6.** Quantitative analysis of M@GM nanoparticle cellular uptake by GL261 glioma cells at different incubation time points (0, 1, 2, 4, and 6 h) using flow cytometry. Data are presented as mean ± SD (n = 3). ***P < 0.001, ****P < 0.0001.

**Figure S7.** Intracellular reactive oxygen species (ROS) levels in GL261 glioma cells after treatment with PBS, M, GM, or M@GM. Data are presented as mean ± SD (n = 3). ****P < 0.0001.

**Figure S8.** Western blot analysis of cleaved Caspase-3 expression following different treatments.

**Figure S9.** Western blot analysis supporting the functional involvement of the Caspase-3/GSDME axis in M@GM-induced cell death. (G1, PBS; G2, M@GM; G3, M@GM + GSDME knockdown; G4, M@GM + Caspase-3 inhibitor; and G5, M@GM + GSDME knockdown + Caspase-3 inhibitor.)

**Figure S10.** Western blot analysis of GSDME-N generation following treatment with free GSDME or M@GM.

**Figure S11.** Quantitative analysis of tumor area in brain sections following different treatments.

**Figure S12.** Immunofluorescence images of Caspase-3, GSDME and GSDME-N in orthotopic glioma tissues after treatment with PBS, M, GM or M@GM. Green signals represent target proteins, DAPI stains nuclei (blue). Scale bar = 100 µm.

**Figure S13.** Immunofluorescence images of ICD markers HMGB1 and CRT in orthotopic glioma sections treated with PBS, M, GM or M@GM. Green signals denote target proteins, nuclei stained with DAPI. Scale bar = 100 µm.

**Figure S14.** Gating strategy for DC analysis of Figure 7a.

**Figure S15.** Gating strategy for macrophages analysis of Figure 7c.

**Figure S16.** Gating strategy for macrophages analysis of Figure 7e.

**Figure S17.** Gating strategy for T cell analysis of Figure 7g and i.

**Figure S18.** Gating strategy for Treg cell analysis of Figure 7k.

**Figure S19.** (a) Gating strategy for IFN-γ⁺CD8⁺ T cells analysis. (b, c) Representative flow cytometry plots and quantitative analysis of IFN-γ⁺CD8⁺ T cells.

**Figure S20.** (a) Gating strategy for Granzyme B⁺CD8⁺ T cells analysis. (b, c) Representative flow cytometry plots and quantitative analysis of Granzyme B⁺CD8⁺ T cells.
